# Short-Range Temporal Interactions in Sleep; Hippocampal Spike Avalanches Support a Large Milieu of Sequential Activity Including Replay

**DOI:** 10.1371/journal.pone.0147708

**Published:** 2016-02-11

**Authors:** J. Matthew Mahoney, Ali S. Titiz, Amanda E. Hernan, Rod C. Scott

**Affiliations:** 1 Department of Neurological Sciences, University of Vermont College of Medicine, Burlington, Vermont, 05405, United States of America; 2 Department of Neurosurgery, University of California Los Angeles, Los Angeles, California, 90095, United States of America; 3 Neurosciences Unit, University College London Institute of Child Health, London, United Kingdom; University of Lethbridge, CANADA

## Abstract

Hippocampal neural systems consolidate multiple complex behaviors into memory. However, the temporal structure of neural firing supporting complex memory consolidation is unknown. Replay of hippocampal place cells during sleep supports the view that a simple repetitive behavior modifies sleep firing dynamics, but does not explain how multiple episodes could be integrated into associative networks for recollection during future cognition. Here we decode sequential firing structure within spike avalanches of all pyramidal cells recorded in sleeping rats after running in a circular track. We find that short sequences that combine into multiple long sequences capture the majority of the sequential structure during sleep, including replay of hippocampal place cells. The ensemble, however, is not optimized for maximally producing the behavior-enriched episode. Thus behavioral programming of sequential correlations occurs at the level of short-range interactions, not whole behavioral sequences and these short sequences are assembled into a large and complex milieu that could support complex memory consolidation.

## Introduction

Previous work has identified a remarkable phenomenon called sleep replay, in which hippocampal place cells fire in sequences during sleep that recapitulate sequences of firing that were repeated during repetitive behavior [1,2]. In addition to sleep replay, there have been multiple reports of sequential firing structure (temporal coding) in the awake state during resting periods while navigating. These observations include ‘forward replay’ [3] where a sequence of firing from previous navigation is reactivated during rest in the same order, ‘reverse replay’ [4], where a sequence is reactivated in the reverse order from previous navigation, as well as reactivations that represent novel trajectories through the environment, which include a bias toward goal-oriented trajectories [5]. These phenomena provide evidence that neuronal firing sequences during burst activity in sleep are biased by observed, largely repetitive, behaviors during wake. However, normal awake experience is extremely complex and highly variable but nevertheless this complex behavior is consolidated into memory. The structure of a neural system that is flexible enough to encode complex experiences, and the systems level mechanisms that underpin this flexibility, remains unknown.

An interesting observation about replay is that the sequences are not always the same (e.g. there is some jitter about the replayed sequence). This could simply be an effect of noise or could be necessary for complex memory consolidation. If every time a neuron fired it were participating in a perfect reactivation of a behavioral firing sequence, then there would be no opportunity for that neuron to participate in any other firing sequence. It is already known that neurons participate in multiple sequences as evidenced by remapping of place cells when environments change (remapping). Because multiple environmental representations are multiplexed in the hippocampus, we hypothesize that sleep replay must be a complex mixture of sequences. We suggest that a network that can flexibly construct hi-fidelity, but not perfect, replay of a measured behavior can also construct sequences reflecting unmeasured behavior. To explore this possibility, we statistically characterize the structure of sequential firing during sleep and then establish that expected, behaviorally related sequences can be identified within this structure. This lends support to the idea that other sequences may also be behaviorally relevant. Specifically, we use a novel decoding strategy based on variable length Markov chains (VLMCs) to characterize the sequential firing of cells during sleep after rodents repetitively ran in a circular track. By modeling the bursting of neurons during sleep as Markov chains we make no explicit assumptions about the relationship between these cells firing activity during behavior and sleep. The Markov chain model characterizes the sequential correlations between cells during sleep and we then inspect the fitted model for behaviorally relevant sequential firing.

We report three key findings. First, we find that short-range temporal correlations between neural bursts are sufficient to explain the correlations in long sequences, indicating that the ensemble is structured in short sequences that are built into longer and more complex sequences, some of which are related to the expected replay sequence. Second, the sequential structures represented by the fitted Markov models are not ‘noisy cascades’ that prefer one or a few distinguished sequences, but rather they support a tremendous number of distinct sequences. Third, we see significant sequential correlations between pyramidal cells that had strong place-specific firing during the behavioral task and hence have clear behavioral sequences and other pyramidal cells that did not.

We suggest that at the system-level our results indicate that the brain is replaying fragments of experience and the dynamics of the ensemble mix these fragments together in a huge variety of ways to consolidate the memory of a huge variety of behaviors.

## Results

Single unit activity from the CA1 region of the hippocampus was recorded from adult rats running in a circular track for 20 minutes (RUN) following which they slept for up to 1 hour (POST). We recorded 5 sessions in 3 animals and retained all pyramidal cells for analysis (Table 1). Analyses were performed on data collected during the entire motionless period after RUN.

**Table 1:** Classical place cell and other pyramidal cell numbers. Previous reports have indicated that approximately 30–50% of hippocampal pyramidal cells are place cells in any given spatial context. In our cohort we measured between 20% and 50%, in accordance with previous reports.

Rat (session)	1 (1)	1 (2)	2	3 (1)	3 (2)
# Classical place cells (# cells)	4 (10)	3 (12)	7 (15)	3 (6)	1 (6)

### Spike avalanches in neural activity during sleep

**Fig 1:**
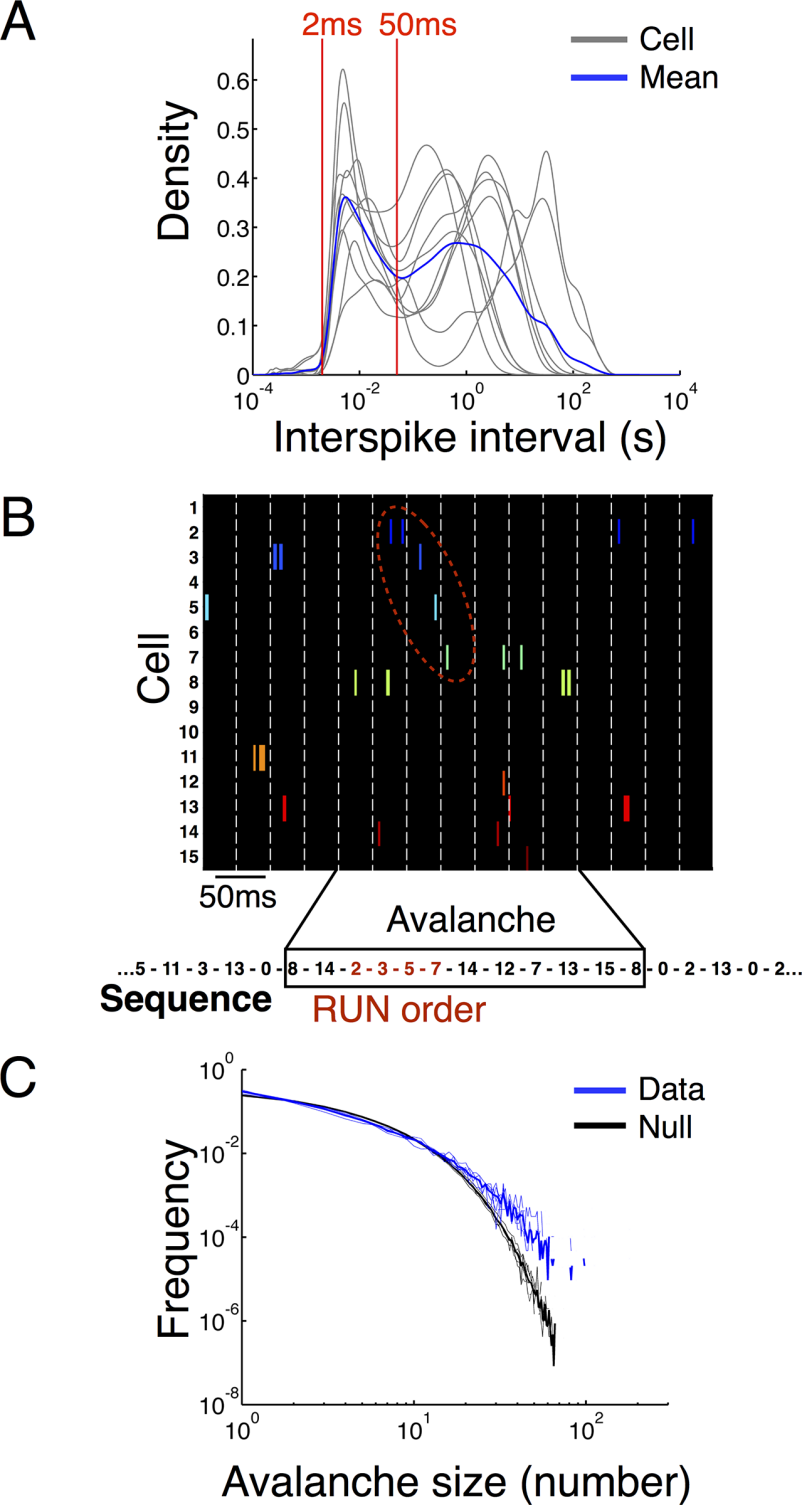
Burst structure of single units and spike avalanches. (A) The distribution of log-interspike intervals during sleep for a representative example (rat 1, session 1) is bimodal demonstrating a fast spiking regime with long gaps (bursting). The distributions for individual pyramidal cells from the session are shown in gray and the population average is in blue. Interspike intervals < 50ms (red line) indicate that two spikes belong to the same burst event. (B) Single unit bursts are correlated in time to produce ‘spike avalanches’ with many units active in a short period of time. An avalanche is defined as a sequence of time bins with spikes from some cell in the ensemble flanked by empty time bins. An example of parsing the burst activity within an avalanche into a discrete sequence is shown below. The boxed sequence is a ‘large avalanche’ that contains a subsequence that is a length-4 match to the RUN sequence (in red). (C) Rank-frequency plots of spike avalanche sizes (blue, population average over sessions in bold) compared to ISI-randomized data (black, average over sessions in bold) show that large avalanches are more common in the observed data than expected at random (all p < 10^−13^, Table 2).

Prior to an analysis of temporal structure during burst firing activity it is important to establish that there is burst firing of pyramidal cells during sleep. Across all of the sessions and animals the distribution of log-interspike intervals (ISIs) is bimodal, with a rapid firing regime (ISI < 50ms) and a ‘long wait’ regime (ISI > 50ms). Each pyramidal cell had a bimodal (log) ISI distribution and the average distribution across all cells shows a clear separation of time scales (Fig 1A). We define single unit *bursts* as sets of action potentials that are separated by less than maxISI = 50ms (c.f. Lee et al. [2]).

Across the neuronal ensembles, these bursts are correlated with each other in time, corresponding to *spike avalanches* where many neurons become simultaneously active [6,7]. We binned the spike trains and found spike avalanches, defined as consecutive time bins with at least one cell firing flanked by time bins with no activity from any cell (Fig 1B; see Materials and Methods and [7]). The timescale for binning was selected by computing the average waiting time for a spike from any cell in the ensemble; a spike avalanche therefore is a set of action potentials preceded and succeeded by a longer than average wait for an action potential from some neuron. These ‘rate-normalized’ time bins are data set dependent and allow for comparing recordings of different numbers of cell and action potentials, as for a fixed time bin recordings with more (fewer) action potentials would have a systematic shift toward larger (smaller) avalanches (c.f. [7,8]). The ‘size’ of a spike avalanche is defined as the number of spikes within the avalanche. Consistent with earlier reports [7], we find that the size distribution of spike avalanches is universal across rats and sessions and has a heavy tail relative to a null distribution of spike trains generated by independently randomizing interspike intervals (Fig 1C). ‘Universality’ means that the distribution of avalanche sizes is the same across all rats and sessions. The ‘heavy tail’ of the distribution means that large avalanches are much more common in the real data than in surrogate data of uncorrelated spike trains; action potentials happen in ‘clumps’ whose sizes are not predictable from what is expected of uncorrelated neurons, even after accounting for the ‘burstiness’ of individual neurons (ISI randomization). To quantify this difference in tails, we defined a *large avalanche* as an avalanche whose size exceeds 99% of all sizes in the null data. All of the measured distributions had significantly more large avalanches than expected under the null model (all p < 10^−13^, binomial test; see Table 2).

**Table 2:** Statistics of avalanche sizes and word lengths. The average number of spikes in a spike avalanche (size) is larger than expected from ISI-randomized null data. The number of large avalanches (>0.99-quantile of the null size distribution; see Materials and Methods) is significantly higher than expected in null data (binomial test). The average number of neural bursts (word length) is also larger than expected in the null distribution. Likewise, the number of long words is significantly larger than expected in null data.

Rat (session)	Mean avalanche size (null mean ± std. err.)	# large avalanches (null expectation, p-value)	Mean word length (null mean ± std. err.)	# long words (null expectation; p-value)
1 (1)	4.81 (4.51±0.02)	345 (105; 3.6e-66)	2.76 (2.60±0.01)	318 (110; 9.8e-60)
1 (2)	4.59 (4.35±0.02)	172 (67; 4.9e-17)	3.33 (3.16±0.02)	158 (67; 8.7e-22)
2	4.96 (4.42±0.02)	713 (255; 1.1e-84)	2.91 (2.60±0.01)	947 (293; 5.2e-205)
3 (1)	5.29 (4.59±0.05)	69 (20; 5.7e-14)	3.49 (3.02±0.03)	68 (22; 1.2e-15)
3 (2)	5.03 (4.67±0.04)	138 (51; 1.2e-16)	3.65 (3.39±0.03)	140 (59; 8.0e-20)

Previous studies of episodic reactivation during sleep have focused on short (~150ms) epochs called ‘sharp-wave ripples’ (SWRs) during which the local field potential (LFP) has high power in the ‘ripple band’ (>150Hz). Many neurons become active during SWRs resulting in long sequences, but it is known that every neuron is also active outside of SWRs [2]. Indeed, there is a smooth gradation between avalanches with few spikes to those with many spikes (Fig 1C), indicating that there is not a categorical difference between the small and large avalanches. Furthermore, the SWR signal in the LFP is in part due to the activity of many cells firing at once and may serve as only a marker for large avalanches, but not their cause. Thus, avalanches are a unit of analysis that is conceptually distinct from firing activity only during SWRs. We note that other authors have used single unit criteria for identifying SWR periods that are essentially equivalent to looking for large spike avalanches (c.f. [2]).

It is known that the firing order of neurons within SWRs contains information about behavior prior to sleep [2]. The time-ordered collection of single neuron bursts within an avalanche is a discrete sequence that describes the ensemble activity during these events (Fig 1B). We parsed each spike avalanche into ‘words’ using a method adapted from Lee et al. [2]. Specifically, for a single neuron we considered spikes separated by less than 50 ms (maxISI) to be a single interval of activity represented by its initial spike time. We encode these activity intervals as *symbols*, e.g. ‘1’ for neuron 1 and so on. The sequential behavior of the full ensemble is then represented as a sequence of symbols, each with a corresponding initial timestamp. To preserve the spike avalanche structure of the ensemble, we only parsed neurons into bursts within an avalanche. Between avalanches, we add a new symbol ‘0’ for each empty time bin to indicate these silent periods in the ensemble activity. The parsed data are a sequence of *burst words* separated by ‘0’s. We call this full sequence the *burst sequence* (Fig 1B). The burst sequence representation of the spiking dynamics is a coarse-graining of the spiking behavior where we ignore the fast timescale rate modulations of the spiking but we capture the latency to first spike for each neuron in the succession of bursts across the ensemble. This allows us to distinguish rate coding from temporal coding. We note that the burst sequence parsing depends on both the bin size for defining avalanches and the maxISI for defining a burst; different choices of these parameters will result in different sequences. However, our choices are natural and data-driven. The universality of avalanche distributions (Fig 1C) results from the rate-normalized time bins (as described above) indicating that an avalanche is a meaningful unit of ensemble activity and by extension that the binning timescale is meaningful. The choice of maxISI is dictated by the burstiness of pyramidal cells, which have a clear bimodal distribution of log-ISIs with maxISI = 50 ms splitting the two modes (Fig 1A).

We define the *length* of a burst word as the number of symbols (with repeats) in the word. In addition to the avalanche size distribution, the length distribution of burst words is also heavy-tailed relative to the null distribution. Analogous to large avalanches, we define *long words* as words with length exceeding 99% of the null distribution. Long words are generated by the true ensemble significantly more often than in null data (all p < 10^−6^, binomial test; see Table 2). Thus, large avalanches are composed of more bursts, not just more spikes per burst.

### Second order sequential structure in spike avalanches

The burst sequence does not, in principle, have to have any statistical structure, i.e. bursts could happen independently according to their burst rates without any preference for neurons to fire in particular sequences. To determine if the burst sequence had nontrivial sequential structure, we fit variable length Markov chains (VLMCs) of varying maximum depth to the burst sequence (see Materials and Methods).

A VLMC is a type of Markov chain, a class of stochastic models of discrete sequences that predict the symbols of a sequence as a function of history of the sequence. A Markov chain is parametrized by a matrix of transition probabilities with rows indexed by an exhaustive set of possible sequence histories (*contexts*) and columns indexed by the possible upcoming symbols (*alphabet*). The probability of occurrence of each upcoming symbol therefore depends on the context. For example, the probability of seeing a ‘2’ in the sequence depends on which symbols are immediately preceding. How much of the history is relevant and how these histories modify the symbol probabilities are learned from the data [9]. The maximum context length is called the *depth* of the model.

Unlike fixed order Markov models, VLMCs allow the lengths of the contexts to vary. In a d^th^-order Markov model for an alphabet of size A, one has to estimate the transition probabilities for each of the *A*^*d*^ possible histories, resulting in a model with *A* ⋅ *A*^*d*^ = *A*^*d*+1^ free parameters. This makes high order models impractical for sequences with even modest alphabet size. The central insight of VLMCs is that not every possible history is equally relevant for describing the sequential structure of the data. Some (potentially long) contexts frequently occur and are highly predictive of the future of the sequence, while others might not contribute predictive power over shorter contexts that generalize them. For example, does knowing that a ‘1’ instead of a ‘2’ happened 6 bursts ago appreciably modify our predictions about the next element of the sequence over and above the prediction we would make knowing only the last 5 bursts? For a general sequence, the answer to this question is nontrivial. VLMC learning algorithms identify the relevant contexts and learn transition parameters for them, solving this problem in a purely data-driven manner. We stress that this context learning is conceptually important even if (as we will see below) the optimal models are low depth; *a priori* a structured sequence could be structured through short- or long-range interactions. Identifying these interactions is critical to understanding the data sequence. Thus, VLMC models can, in principle, capture long contexts, say length 5, without the burden of having to fit a full 5^th^-order Markov chain [9] and are therefore highly flexible models for sequential data.

**Fig 2:**
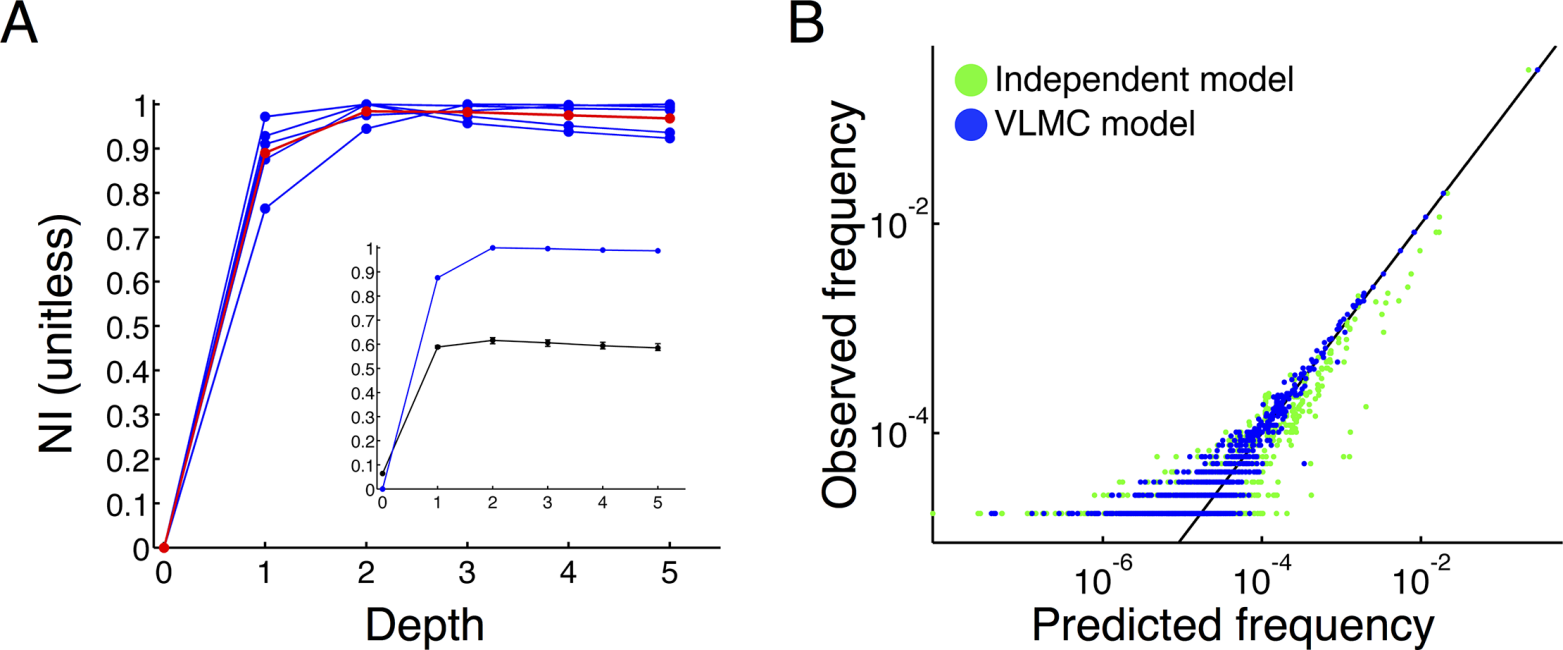
Second order statistical structure in population bursts. (A) Cross-validation of VLMC models demonstrates that the optimal memory depth for predicting the future of the burst sequence is d = 2, where the normalized sequential information (NI) approaches it asymptote of 1. Blue curves show NI as a function of model depth for each rat and session. The red curve shows the population average. Note that ~90% of the sequential structure is captured at depth d = 1 (first-order Markov model) with an improvement (~10%) at depth d = 2, after which point the VLMC models saturate. (Inset) NI as a function of depth for the fitted model (blue, Rat 1, session; all sessions are similar) compared to models fitted on ‘null sequences’ that have had neural bursts randomly permuted (black) shows that that sequential structure between neurons is a significant feature of the burst sequences. The null sequences preserve the avalanche structure, which is captured as a first-order Markov chain, but the null models saturate significantly below NI = 1 (error bars show the min and max NI for 100 permutations of the burst sequence). (B) The VLMC models predict the frequency of occurrence of each distinct burst word in the burst sequence. Log-log plots of observed versus predicted frequencies for a representative example (Rat 2) of the burst words demonstrate that the fitted VLMC model outperforms the uncorrelated model. Each point corresponds to a unique word from sleep and the color corresponds to the fitted model (blue) or independent model (green). A perfect match between the predicted and observed frequencies would correspond to points along the black line (y = x). Note that at the low frequency end the sampling error results in larger deviations in the graph from the identity line.

We used 10-fold cross-validation to avoid overfitting to the burst sequence and to assess the maximum depth at which the statistical structure is captured. We define the *normalized information*, NI, as the fraction of total sequential information available in the data sequence captured by the depth-d models (see Materials and Methods for mathematical definitions). NI has a value of zero if a model has no sequential structure at all (i.e. independent random sampling) and has a value of one if it is the optimal model. We see that across all rats and sessions the VLMC models saturate at depth 2 meaning that the best prediction of the upcoming symbol in the sequence depends most strongly on the previous two symbols. On average, first-order Markov chains captured around 90% of the sequential information and the second order structure captured a further 10% (Fig 2A). This saturation at depth 2 is potentially a feature of the measuring a small subset of neurons in the full hippocampus and it may be that measuring larger ensembles would reveal longer-range sequential structure. Indeed, the probabilities in the VLMC models are an estimation of the sequential correlations between the measured neurons and these correlations are certainly a function of the firing of other, unmeasured neurons. Nevertheless, a statistical description of a superset of the measured ensemble will have to produce the observed sequential correlations in the measured ensemble, but interleaved with bursts from other unmeasured cells. Cross-validation allows us to tune the fitted model to the robust correlations in the measured ensemble without overfitting.

The above shows that the burst sequence is sequentially structured, but there are two types of symbol in the sequence, symbols representing bursts (e.g. ‘1’, ‘2’, ‘3’, etc.) and the symbol representing silence (‘0’). To see if the fitted model contains nontrivial structure between neural bursts and not just structure related to the population bursting of avalanches, we scrambled the burst sequence by randomly permuting the bursts within the sequence but fixing the ‘0’s. This preserves the burst rates of each neuron and all of the temporal structure between silence and bursting, but reduces all sequential correlations between bursts to noise. We fit VLMCs to these scrambled sequences. The models fitted on the scrambled sequences achieve only a fraction of the normalized information of the true data sequence (Fig 2A, inset). We emphasize that the scrambled sequences are structured; they have significant correlations due to the avalanche structure of the burst sequence, but those correlations alone do not account for all of the sequential information learned by the fitted models on the true data sequence. For subsequent analyses we used VLMCs of maximum depth 2 and we refer to this model as the *fitted model*. Note that because VLMCs learn the relevant contexts, which have variable length, they are buffered against model misspecification, e.g. while fitting a depth-2 model on first-order data the algorithm ‘prunes’ the contexts back to first-order (for a detailed discussion of VLMC model learning see Begleiter et al. [9]).

The VLMC models make predictions of the rates of occurrence of subsequences within the burst sequence [9]. To visually assess the model fit, we computed the expected number of occurrences of each unique observed burst word under the fitted model and compared the predicted frequencies to the observed frequencies of the words (Fig 2B, blue points). The independent model, which assumes the same symbol frequencies but no sequential correlations, makes much larger errors in predicting the word frequencies (Fig 2B, green points) demonstrating that sequential correlations are significant features of observed burst sequence. Thus, the VLMC models are capturing the longer-range sequential structure within the burst sequence, even though they only explicitly account for interactions across three consecutive letters (second order model). In other words, the short-range temporal interactions of the ensemble are sufficient to describe the long-range sequential structure.

### Replay of behavioral sequences

**Fig 3:**
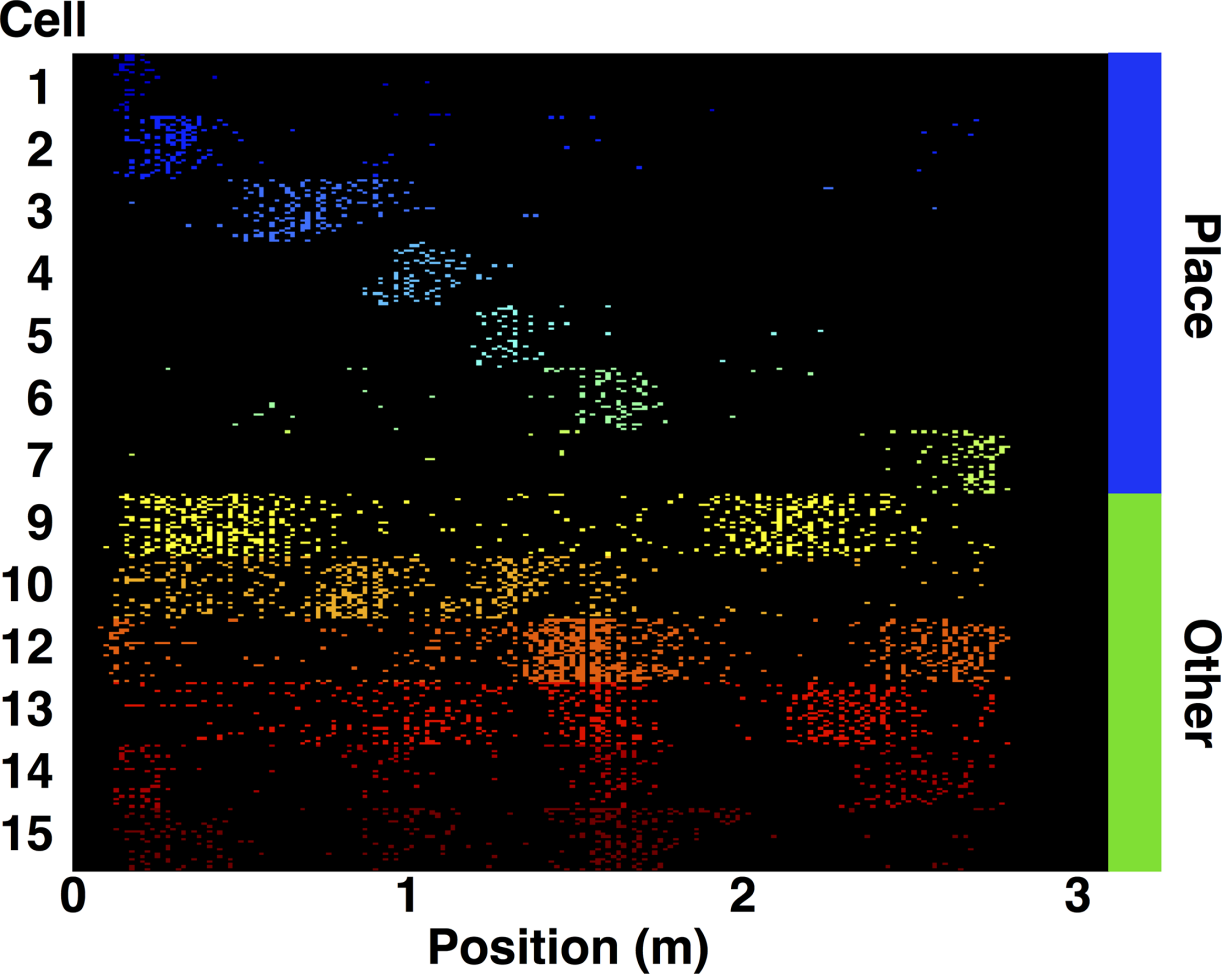
Spatial variation of pyramidal cells firing during RUN. During behavior rats ran in a 1m diameter circular track for 20 minutes for food reward. Raster plots of pyramidal cell firing show the spatial rate modulation for the measured cells. Angular position along the track is binned into 200 bins (~16cm of arc). Each cell has a color and spatial bins are colored by whether or not the cell spiked within that bin during a trial. Trials are stacked on top of each other by cell, so each set of rows of one color represents the spiking of a single cell over all trials. We only display data from trials in one direction around the track (the direction with the most turns in the session) and only cells that had > 50 spikes for such trials. The firing rate of measured pyramidal cells varied as a function of angular position along the track (Rat 2, but all sessions are similar). Some cells had single place fields indicating a strong place preference for firing (classical place cells, blue bar at right). Other cells showed firing rate variation as a function of angular position, but with multiple firing peaks (other pyramidal cells, green bar at right). The ‘RUN sequence’ is defined as the order of firing of the classical place cells along the direction of the track. In this case, the RUN sequence is 1-2-3-4-5-6-7, as the first seven cells are classical place cells that fire in that order as the rat runs along the track. The classical place cells have largely nonoverlapping place fields, while the other pyramidal cells cofire with multiple place cell and each other and do not provide obvious sequential episodes during behavior like the classical place cells.

Prior to sleep, the rats ran in a circular track for a food reward. During behavior the firing rates of pyramidal cells vary as a function of the rats’ position. The position-dependent firing can be highly localized in a single firing field or more diffuse with multiple firing fields (Fig 3 and S1–S4 Figs; Table 1). We call these two pyramidal cell subtypes *classical place cell*s and *other pyramidal cells* respectively. It is unknown what hippocampal inputs are relevant for determining the spatial firing rates of individual pyramidal cells, but it is known that these rates depend on position, experimental context [10–12], and the cell assemblies to which the cell belongs [13]. Classical place cells fire in sequences during spatial navigation and repetitive behavior leads to repetitive activation of particular sequences, which allows us to define a behavioral ‘RUN sequence’ associated to the experimental behavior (Fig 3 and S1–S4 Figs). We define the *RUN sequence* as the sequence of classical place cells ordered by their firing order as the rat traverses the track; in Fig 3 the RUN sequence is 1-2-3-4-5-6-7. We note that the RUN sequence is defined exclusively from behavior and is completely independent of our analysis of firing during sleep.

The firing sequence of classical place cells during behavior is known to be related to the burst activity during sleep as it has been shown that sequential firing during sleep is biased to produce partial matches to the RUN sequence. Furthermore, sleep replay has been observed with similar numbers of recorded cells as in the current study [2]. Four of five sessions representing all three rats had multiple place fields allowing for defining a RUN sequence and looking for RUN sequence enrichment during sleep.

Consistent with reports of hippocampal replay, we find that the RUN sequence observed in each rat and session is more probable under the fitted model (Tables 3 and 4) than under a comparator model, the *avalanche independent model* (AIM), that has no sequential correlations between neurons but does preserve the burst rates and avalanche structure (see Materials and Methods). We define the *likelihood ratio* of a sequence as the ratio of the probability of the sequence under the fitted model to the probability under the AIM. When the likelihood ratio exceeds 1 the sequence is *enriched*, otherwise it is *depleted*. The likelihood ratio quantifies the extent to which the sequential structure of the fitted model is biased to produce the particular sequence relative to the AIM. A likelihood ratio of 2 indicates that the correlations in the data make the appearance of that sequence twice as likely as would be expected in sequentially uncorrelated data. The likelihood ratio for the RUN sequence was greater than one for all sessions, indicating that the RUN sequence is enriched in the sequential structure of sleep firing.

**Table 3:** Replay of RUN-order sequences. During behavior place cells fire in sequences that are replayed during sleep. The fitted VLMC models encode the sequential correlations between cells during sleep and predict the probabilities of the RUN sequences and their subsequences (RUN-order sequences). The majority of these likelihood ratios indicate that they are enriched relative to avalanche independent model. However, the raw likelihood ratios do not directly attest to the statistical significance of this enrichment. We assessed significance using the z-score of the likelihood ratio computed using the values from 10-fold cross validation. These z-scores incorporate the ‘jitter’ in the model fitting and provide a strong signature of statistical significance of the likelihood ratio. The significance level was set at p < 0.05 after Bonferroni correction for multiple hypothesis testing. However, we note that the likelihood ratios for all sequences are correlated, so this significance level is stringent. The full RUN sequence was significantly enriched in 3 of 4 sessions (representing all three rats).

Rat (session)	# Enriched (Significant) RUN-order sequences (/ Total)	Full RUN sequence enriched (significant)?
1 (1)	9 (5 / 11)	Yes (Yes)
1 (2)	4 (2 / 4)	Yes (No)
2	91 (55 / 120)	Yes (Yes)
3 (1)	4 (2 / 4)	Yes (Yes)

**Table 4:** Enrichment of the RUN sequence during spike avalanches. The RUN sequence occurs at different rates in different Markov chain models (avalanche independent, true, and RUN-optimized; columns 2–4 respectively). In the fitted model the RUN sequence occurs at a rate between 1.3- and 2.2-fold higher than under the temporally unstructured avalanche independent model demonstrating that there is a statistical enrichment for the RUN sequence in the burst sequence (column 5). The true enrichment is only a small fraction of the amount possible given the burst rates. Under the RUN-optimized model, the RUN sequence occurs at rates between 60- and 2x10^8^-fold over the avalanche independent model (column 6). This demonstrates that the fitted model is not producing the RUN sequence close to the theoretical maximum rate.

Rat (session)	Independent rate, r_AIM_ (occurrences per symbol)	Fitted rate, r_fit_ (occurrences per symbol)	Maximal rate, r_max_ (occurrences per symbol)	r_fit_ / r_AIM_	r_max_ / r_AIM_
1 (1)	1.68e-07	2.49e-07	1.04e-03	1.48e+00	6.18e+03
1 (2)	7.11e-07	9.34e-07	3.04e-03	1.31e+00	4.27e+03
2	4.03e-13	8.71e-13	6.87e-05	2.16e+00	1.70e+08
3 (1)	1.92e-04	2.98e-04	1.15e-02	1.55e+00	5.97e+01

However, the likelihood ratios, as well as the computed RUN sequence probabilities under the Markov models, are functions of the training burst sequences, which inevitably have sampling noise. We can assess statistical significance of a likelihood ratio by considering the ‘jitter’ in the model fitting during cross-validation. We assign z-scores and p-values to sequences based on the variance of the ratios when some training data is held out (see Materials and Methods). Note that p-values for different sequences are correlated; so multiple hypothesis testing corrections are likely to be conservative. The z-scores for 3 out of 4 sessions representing each of the rats were statistically significant.

In addition the RUN sequence, we define *RUN-order sequences* as sequences of place cells that occur in the behavior order but with gaps. For example, 1-3-4 is a RUN-order sequence for the RUN sequence 1-2-3-4-5-6-7 in Fig 3. These sequences allow us to ask whether partial reactivations of the RUN sequence are enriched as well as the full sequence. Analyzing RUN-order sequences also allows us to answer whether we would have been able to identify RUN enrichment if we had not measured all of the cells we did measure. Conceptually this is an important positive control because every experiment is limited in how much of the system it measures.

**Fig 4:**
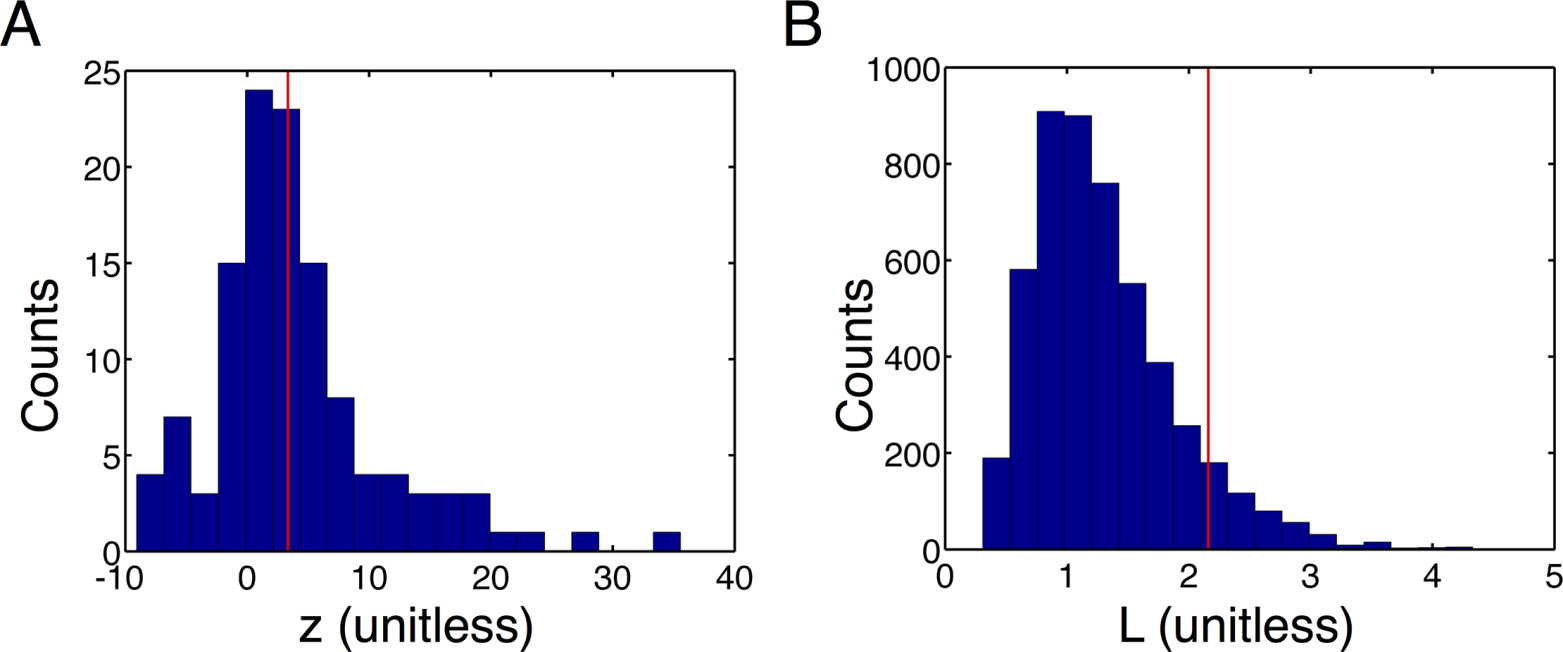
Enrichment of behavior related sequences. (A) The histogram of standardized log-likelihood ratios of all ‘RUN-order’ sequences for Rat 2 shows enrichment of subsequeces of the RUN word. The RUN sequence for Rat 2 was 1-2-3-4-5-6-7 (see Fig 3), and RUN-order sequences are any sequence in which cells fire in this order, but with gaps (e.g. 1-3-5-7). There are 120 such sequences and 55 of them are significantly enriched (see Table 3) indicating significant enrichment for sequences in RUN-order. The vertical red line indicates the significance level p < 0.05 with a Bonferroni correction for multiple hypothesis testing. (B) The histogram of likelihood ratios of all 5040 permutations of the RUN sequence for Rat 2 shows that the RUN sequence has a likelihood ratio in the 9^th^-percentile of this distribution (vertical red line), indicating that it is strongly enriched relative to other comparable sequences in the ensemble firing. Note that this distribution shows both up- and down-regulation of place cells sequences in the fitted model indicating that the sequential structure in the fitted model does prefer for place cells to fire in certain orders and not others. Thus, the enrichment of the RUN sequence cannot be attributed to a general up-regulation of arbitrary sequences of these place cells.

We computed z-scores for all RUN-order sequences. For all sessions approximately 50% of all RUN-order sequences were significantly enriched (p < 0.05 Bonferroni correction for multiple hypothesis testing; Table 3). As described above, these significant enrichments include the full RUN sequence in three out of four sessions. (Rat 1, session 2 had a 3 cell RUN sequence, two length-2 subsequences of which are significantly enriched.) The histogram of z-scores for the 120 RUN-order sequences for Rat 2 shows that many (55/120) of those sequences as significantly enriched in the fitted models (Fig 4A). Note that there are also significantly depleted sequences in Fig 4A (large negative z-scores), indicating that the enrichment of the RUN-order sequences is not due to a general up-regulation of arbitrary place cell firing sequences.

The enrichment of the RUN-order sequences shows that the sequential structure during sleep produces the RUN-order sequence at rates higher than would be expected if the neuronal bursts within an avalanche were completely uncorrelated. Rat 2 had a long sequence of classical place cells (7/15 cells) that could be used to further explore the enrichment of the RUN sequence. We computed the enrichment of all 5040 permutations of the length-7 RUN sequence. (Note that these 5040 sequences are a tiny subset of all 15^7^ = 170,859,375 possible length-7 sequences from the 15 measured cells.) The RUN sequence is in the top 9% of likelihood ratios for the permuted sequences showing that it is among the most strongly enriched sequences that can be built out of the 7 place cells (Fig 4B). Note that this distribution of likelihood ratios for permutations of the RUN sequence does not constitute a null distribution nor does the 0.09 quantile of the RUN sequence within this distribution constitute a p-value. The null model is the AIM and the RUN sequence as well as many of the permuted sequences has a likelihood ratio exceeding one, indicating enrichment. Also note that, as with Fig 4A, there are many likelihood ratios below 1, indicating depletion of certain sequences. Thus, the up-regulation of the RUN sequence and many permutations of it cannot be ascribed to a general up-regulation of all place cell sequences.

### RUN optimal Markov chains

The trained behavior of running in a circular track for food is clearly a salient experience for the rats and the RUN sequence is enriched during sleep, but it is only one of many such sequences. Thus, the RUN sequence is not occurring at the theoretically maximum rate. It remains unclear, however, what that maximum rate would be and how the true rate compares to this maximum rate.

We constructed first-order Markov chains that maximized the rate of occurrence of the RUN sequence subject to the constraint that the burst rates of each neuron are equal to the observed rates. In effect, we identified the Markov chains that produce the maximum amount of RUN sequence possible given the burst rates. This optimized model allows us to characterize the capacity of a hypothetical ensemble with the measured burst rates and avalanche structure to produce the RUN sequence. This model describes the scenario where a particular spatial memory is prioritized over all other memory encoded by these cells and sleep is constrained to rigidly generating that specific cascade whenever any place cell fires. We compared this maximum rate (*r*_max_) to the rate under the fitted model (*r*_*fit*_) and the AIM rate (*r*_*AIM*_) and we note that, while in all cases the RUN sequence appears more often under the fitted model than under the AIM, the fitted model produces the RUN sequence at only a tiny fraction of the rate possible for the measured burst rates (Table 4).

Note that the rates predicted by the models are inversely related to the length of the sequence and these rates are expected to go to zero as the sequence size grows. This is because for a larger number of cells there are more options for transitions. Thus we expect the discrepancy between the fitted rate for any sequence and the model optimized for that sequence to grow with the number of cells. Nevertheless, the RUN-optimal models characterize the capacity for the ensembles to produce the RUN sequence and provide a check for how well the fitted models approximate the perfect RUN cascades. These rates demonstrate that the ensembles are not simply ‘noisy’ RUN sequence generators during sleep and the place cells participate in many sequences other than the RUN sequence.

### Sequential structure between classical place cells and other pyramidal cells

**Fig 5:**
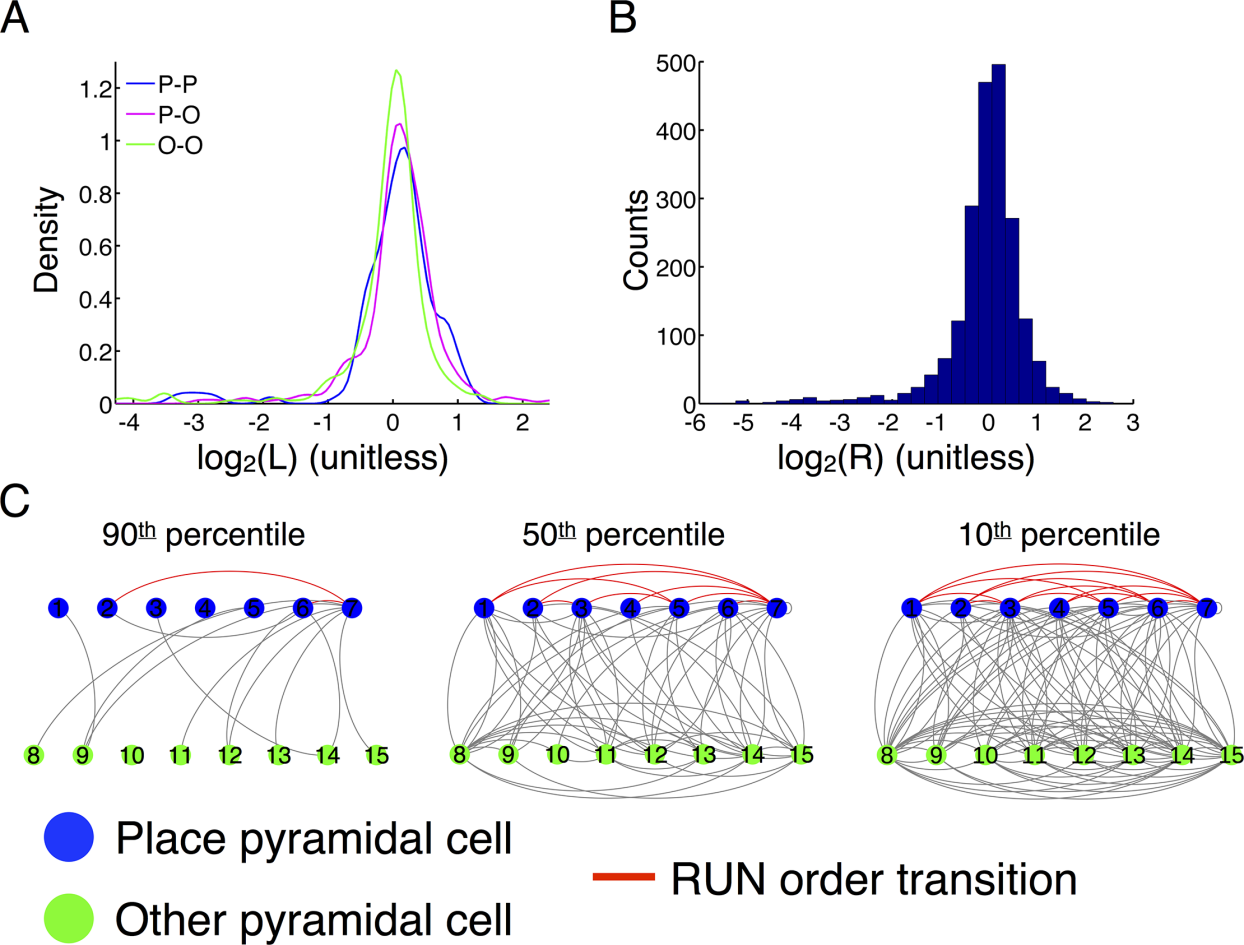
Interactions between classical place cells and other pyramidal cells. (A) The densities of likelihood ratios for place-place, place-other, and other-other digrams (2-element sequences) qualitatively show no preference for classical place cells to fire with other classical place cells (blue density) or other pyramidal cells with to fire with other pyramidal cells (green density) versus firing together (magenta density). Permutation testing shows that none of the distributions is significantly different (all p > 0.05, Kolmogorov-Smirnov statistic for 1000 random permutations of cell labels). Densities are shown for likelihood ratios pooled for all rats and sessions. (B) The histogram of conditional log-likelihood ratios for the fitted models (pooled for all rats and sessions) shows that the historical context of the burst sequence modulates the bursting rates of the other neurons in the ensemble between 64-fold below and 8-fold above background with typical values between ±2-fold of background. (C) The set of enriched transitions in the first-order Markov chain fit to the burst sequence can be viewed as a network of transitions between cells, where an arc is placed from one cell to another if the bursting of the source cell up-regulates the firing rate of the target cell. This network is directed; left-handed arcs from a node represent paths leading away from a node. Paths in this network represent sequence of transitions that are enriched in the temporal structure of the burst sequence. We show the network for Rat 2 for varying thresholds within the rate modulation distribution (see panel B; node labeling corresponds to Fig 3). All rats and sessions look similar. Note that the full network of enriched transitions is densely interconnected (a ‘hairball’) indicating that each transition is part of a complex milieu of possibilities that generates a tremendous variety of sequences during sleep. Furthermore, consistent with (A), classical place cells and other pyramidal cells are highly interconnected with each other. We highlighted the RUN-order transitions (red arcs). Many of these enriched transitions occur in the top 50% of all enriched transitions, but the network highlights graphically the extent of the sequence diversity that is enriched in the fitted model.

Pyramidal cells that are not classical place cells are typically removed from sequence-based analyses of sleep replay because of their complex spatial structure, whereas classical place cells yield distinct sequences of firing that can be expected as replay events [2]. Moreover, their distinction during behavior suggests that other pyramidal cells may play a fundamentally different functional role from classical place cells. Nevertheless, all pyramidal cells are bursty during sleep (Fig 1A). The sequential structure during sleep indicates that *digrams* (2-symbol sequences) of firing history are predictive of the upcoming neuron to fire. These digrams contain both classical place cells and other pyramidal cells, but they do not all appear with equal frequency. Some digrams are relatively common, while others are rare. We computed log-likelihood ratios for digrams under the fitted model and the AIM. Normalizing by the AIM allows for determining which digrams were enriched purely by the temporal structure between neurons in the fitted model and not because of the background firing rates of the neurons, which vary over two orders of magnitude (data not shown). If place cells preferentially fired in sequences with place cells, then we would see a rightward skew in the distributions of the place-place density relative to the place-other density, and likewise for other pyramidal cells. However the three densities are statistically indistinguishable (Fig 5A, all p > 0.05, Kolmogorov-Smirnov statistic for 1000 random permutations of cell labels). Indeed, the sequential structure is mixed between both types of cell. Because a digram can only be enriched if that transition occurs more often in the true data than under the AIM, this demonstrates that classical place cells and other pyramidal cells are predictive of each other’s bursting within an avalanche and therefore correlated.

### Networks and enriched sequences

The VLMC models encode the temporal structure of the burst sequence as a set of transition probabilities from each context (digram) to each possible next burst. We compared the transition probabilities of the fitted models to the AIM by taking log ratio of each transition probability in the fitted model to the corresponding probability in the AIM. This yields a rectangular *conditional log-likelihood matrix*, R, which encodes how each context (indexing a row) up- or down-regulates the burst rates of each neuron relative to the uncorrelated AIM (see Materials and Methods). Analogous to the likelihood ratios for sequences, a transition for which this log ratio exceeds 0 is an *enriched transition* otherwise it is a *depleted transition*. These values vary from 16-fold below background (i.e. some contexts suppress the firing of some neurons) to 4-fold above background with typical values lying between ±2-fold over background (Fig 5B).

The set of enriched transitions can be viewed as a network where nodes are states of the Markov chain and edges represent enriched transitions. This ‘enriched network’ encodes all enriched sequences; by construction every path in this network represents a sequence of transitions that occurs more often under the fitted model than the AIM. Fig 5C shows a representative example (Rat 2), however all sessions are similar. For clarity of display, we have used the first-order Markov model so that nodes represent neurons. It is immediately clear that the network is highly interconnected, with a tremendous number of edges indicating that each burst from a neuron modulates the immediate firing propensities of many other neurons in the ensemble. In addition, edges exist between both classical place cells (blue nodes) and other pyramidal cells (green nodes) indicating that these cells are functionally interacting to produce the observed burst sequence. While classical place cells and other pyramidal cells have potentially distinct functional roles, there is very little distinction between them in the networks of enriched transitions. In particular, the distribution of weighted node degree for the two classes of cells is not significantly different (data not shown). This suggests that each class of neuron is interacting with a similar fraction of the network.

As expected from the enrichment of RUN-order sequences, many of the RUN-order transitions between classical place cells are enriched transitions in the fitted VLMC models. We have highlighted these transitions within the network (Fig 5C, red arcs). Thus, many links from the behavior sequence are enriched during sleep, but each transition takes place within a large milieu of enriched transitions demonstrating that the RUN sequence is among many other sequences enriched during sleep. The RUN sequence is therefore privileged in the sense that it is enriched over the background firing rates, but it is only one of many such sequences.

### Clustered contexts and the next element of the sequence

The second order structure in the Markov chains indicates that the history of bursting, i.e. the previous digram, is the relevant context for predicting the next neuron to burst in the sequence. For example, suppose that the two most recent bursts are the sequence σ = 1–2. The VLMC model encodes the probability for the ensemble to transition to the next symbol given that the history, P_fit_(s|σ). This conditional probability encodes how the firing history of the ensemble modulates the firing rates of neurons. We can compare these rates to the AIM, which has no sequential structure between neurons, to quantify the up- or down-regulation of a transition in the fitted model relative to the baseline firing rates. Specifically, we compute *conditional log-likelihoods* for each transition, which are naturally structured as a rectangular matrix with contexts (digrams) indexing rows and symbols indexing columns (see Materials and Methods for mathematical formulae). We denote these matrices by R.

**Fig 6:**
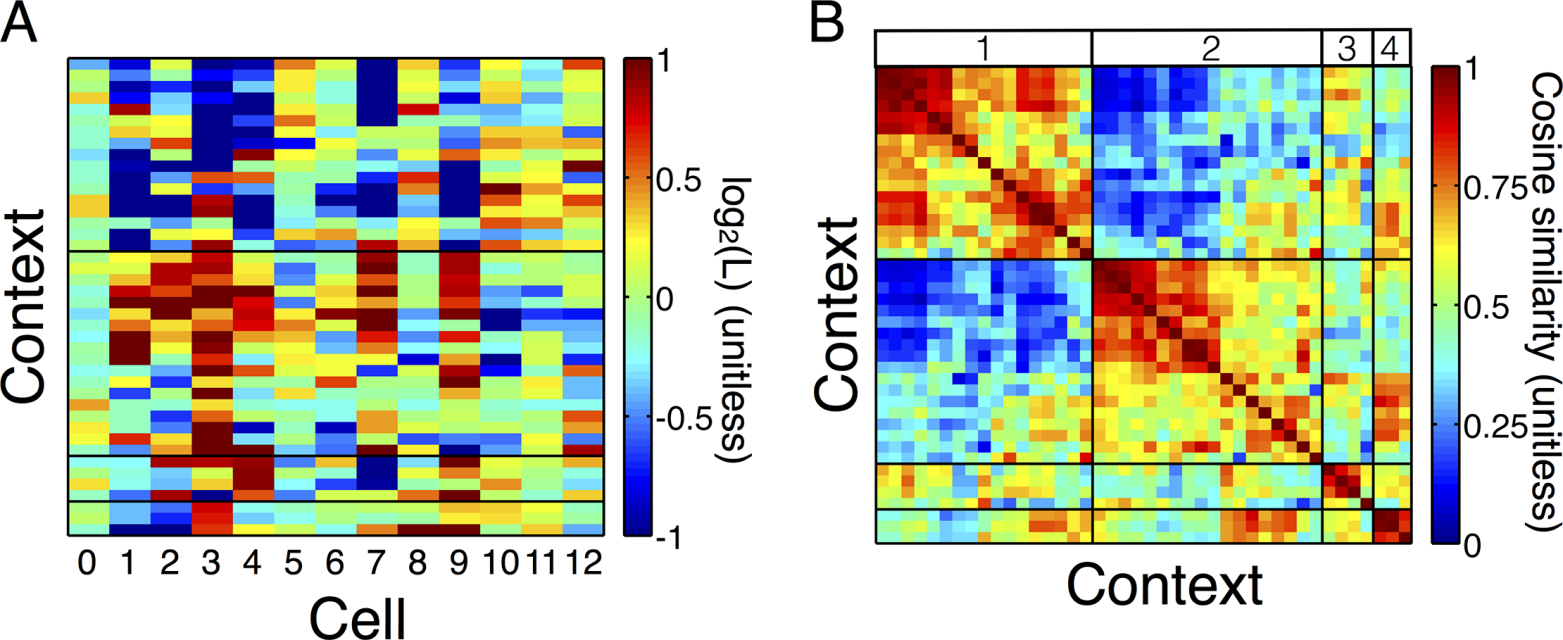
Clustering of VLMC contexts. The historical context of firing modulates the probability of firing for the cells in the ensemble. The strength of this modulation can be measured as a ‘conditional log-likelihood ratio’ comparing the fitted model to the avalanche independent model. These ratios can be organized into rectangular matrices, denoted by R, with contexts in rows and symbols in columns (see Materials and Methods). (A) Heatmap of the R matrix of a representative example (Rat 1, session 2) has clusters of contexts that similarly modulate the ensemble (black lines). The color scale represents the log-likelihood ratio between the fitted model and the AIM; red indicated up-regulation, blue indicates down-regulation. We have set the color scale between -1 (2-fold down-regulation) and +1 (2-fold up-regulation) to clarify the clustering structure, but note that these values have greater variation (Fig 5B). In Rat 1, session 2, clusters 1 and 2 strongly differentially regulate cells 1,3 7, and 9. This indicates that the cells represented in the contexts from different clusters form cell assemblies of coactivating cells. Note however that there is wide variation within each cluster indicating that each context indeed uniquely modulates the ensemble. (B) To visualize the clustering, we computed the cosine similarity matrices between pairs of contexts in the R matrices. These similarity matrices encode how similarly two contexts modulate the ensemble. A value of 1 indicates that the contexts make identical predictions about the upcoming neuron to fire, while a value of 0 indicates that the contexts are extremely dissimilar. The similarity matrices have clear block structure indicating that the clustering in (A) is a strong feature of the R matrices (labels of four prominent clusters shown at the top of the similarity matrix).

Heatmaps of the R matrices show that the contexts cluster into groups according to how they predict the future bursting of the ensemble, i.e. how they modulate the instantaneous firing rates of the neurons in the ensemble. Fig 6A shows a representative example, but all sessions are similar (S5–S8 Figs). Two contexts are considered similar if they similarly regulate the ensemble (i.e. if their corresponding rows in R are similar). We quantify this similarity by computing the cosine of the angle between the row vectors or R corresponding to each pair of contexts (*cosine similarity*, see Materials and Methods). Hierarchical clustering shows that these pairwise similarities between the rows of R have distinct clusters of contexts that similarly modulate the ensemble (Fig 6B). This clustering indicates that these multiple histories share similar futures, in that within a cluster each context is making similar predictions about which neurons are more or less likely to be the next to burst. This clustering is akin to how hippocampal networks are theorized to partition themselves into functional groups called cell assemblies [13–15], or groups of neurons with structured coactivation that potentially encode memory. In this case, different sequential contexts precede futures with similar patterns of enrichment and depletion of particular neural bursts. Independent of the mechanisms by which cell assemblies are formed and activated, the fitted models show that measured ensembles are structured in such a way that certain cells preferentially fire after certain contexts, while the context clustering shows that multiple contexts share their preferences, indicating an robust evolution toward particular patterns of coactivation. This suggests that the sequential structure in the burst sequence is, in part, related to the functional organization of the hippocampus.

## Discussion

### Behavior, memory, and the criticality hypothesis

Sleep replay is postulated to be a phenomenon at the center of memory consolidation [16–19] and appears to be a single unit correlate of the reactivation of episodic memory that is subjectively experienced by humans during sleep [20]. The complexity of daily behavior that is encoded into memory requires a neural system that can capture regularities across disparate environmental exposures encoded by the same set of cells and can integrate those exposures with each other into coherent memories and useful insights that link the memories. A model of this consolidation process is that temporally structured, ‘episodic’, sequences of neural firing get ‘imprinted’ into the system during behavior and those sequences are repeated during sleep to either strengthen the connections within the hippocampus that encode that memory or to transmit a high fidelity copy of the memory to downstream cortical stores of long-term memory. However, work in humans and rodents has shown that memory consolidation does not function simply to strengthen memory, but rather to integrate memory into preexisting associative networks by extracting the ‘gist’ of the experience and to generate insight across multiple experiences [20]. It is unknown how the hippocampus manages the trade-off between high fidelity reactivation of distinct episodes and generalization across similar, but not identical, episodes. We provide evidence that, at the system level, short-range interactions are biased by behavior and that these short-range interactions are summed into long sequences, some of which are behaviorally relevant.

In principle, sequential correlations between bursts within an avalanche could have been trivial. Instead, we see enrichment for certain transitions, including those that generate replay sequences related to the RUN sequence (Figs 4 and 5C). We also observe depletion of other transitions. Our analysis shows that, in the ensembles measured, the optimum depth for modeling burst sequences during sleep is two, meaning that each burst can only directly influence the bursting of a cell at most two bursts in the future. Mathematically however, a depth-2 model can have a variety of dynamical properties. They can be strict cascades, where the previous two bursts strictly determine the future, or they can evolve with high probability to many possible symbols from each context. The fitted VLMCs are the latter (Figs 5C and 6). Thus, we provide direct evidence that ensemble activity during sleep is a complex mixture of sequential activity that is largely constructed out of short sequences, fragments of experience, that are pieced together, analogous to a language in which letters are joined in coherent ways to make words. This augments the observation of replay by placing replay events within a complex context of multiple enriched sequences.

The structure of avalanches in neural networks has been studied extensively in connection to the criticality hypothesis of neural dynamics [6]. At criticality, a neural network displays avalanches that span many orders of magnitude in size and duration. We show that spike avalanches in the hippocampus during sleep are distinguished from null data both in their size (number of spikes) and in the lengths of the burst words contained within them (Table 2). The existence of large avalanches and long words raises the question of whether it is adaptive for the brain to produce these long words. In our experiments, the long words in the burst sequence each appear only once during the entire sleep session. Meanwhile, the short-range statistics of these words are enriched for behavior. This suggests that the diversity of sequences generated by the avalanche dynamics during sleep is functioning to integrate all of these short sequences together in novel ways. This may be related to insight generation, the process of combining disparate pieces of information into a unified framework, a process known to be sleep-reinforced in humans [20]. Thus, the short-time interactions within the neural sequences appears as a mechanism for mediating the tradeoff between needing to imprint specific sequences (memory strengthening) and generating novel associations between memories (insight); behavior enriches certain short sequences, while the avalanche dynamics mix these pieces together in long events. More often than expected at random these mixtures will decode to RUN replay event. Although the only behaviorally relevant sequence that we have formally studied is the RUN sequence, inspection of the enriched networks shows that reverse replay transitions are also enriched in our models (Fig 5C).

### Classical place cells vs. other pyramidal cells

Under the classic criteria [21], a cell is a ‘place cell’ if it has 1) a visually identifiable place field, 2) the place field contains at least 9 contiguous spatial pixels and 3) the spatial coherence is greater than 0.3. These cells are typically selected for sequence-based replay analyses, while cells with low spatial coherence or multiple place fields are discarded, as single firing field place cells yield clear ‘episode sequences’ during behavior that can be identified as replay during sleep. The Markov chains we constructed, in contrast, are composed of bursts from all pyramidal cells measured during behavior. Nearly all pyramidal cells showed rate modulation as a function of position (Fig 3 and S1–S4 Figs), but most (63%) did not localize strongly in a single firing peak (Table 1). Thus, their firing is position dependent, but not to the extent of the distinct single field classical place cells. While other pyramidal cells do not show spatially localized firing during behavior, they are bursty during sleep (bimodal log-ISI distributions; Fig 1A) and become active in concert with the other cells in the measured ensemble. Thus, even though these cells do not undergo simple sequential firing during the experimentally measured behavior, they do fire within spike avalanches during sleep and are surely involved in behavioral encoding.

The hippocampal spatial code is a *population code*, i.e. the neural representation of position is distributed across multiple place cells. Standard tools for analyzing place cell ensembles are Bayesian decoding techniques that predict the location of the rat based on the ensemble firing pattern of place cells by inverting the spatial encoding using Bayes’ rule [22,23]. The trained decoding model is then used to interpret the sleep firing of the same ensemble and various properties of the decoded patterns are observed [24,25]. Bayesian decoding methods do work for pyramidal cells with multiple place fields (our ‘other pyramidal cells’); indeed, any spatial modulation of the firing rate of a cell provides some information about where the rat is. However, Bayesian decoding methods are supervised techniques and cannot be used in situations where it is unknown what features, e.g. a location in space, the neurons represent. Indeed, it is known that pyramidal cells that have high spatial coherence in one arena are often not classical place cells in other arenas [10–12]. Pyramidal cell ensembles therefore likely retain multiple features of the rat’s awake experience, presumably even features that are not under experimental control. If sleep replay of awake experience is a generic phenomenon used for all hippocampus-dependent memory consolidation it must be a complex milieu of memories supported on a shared neural substrate. Sequences that are coherent reactivations of traversing one enclosure could look nonsensical when interpreted as possible reactivations of sequential firing in another enclosure.

One of the virtues of sequence-based decoding of sleep replay is that it works with relatively modest numbers of measured cells as in the experiments reported here (c.f. [2]). If a set of place cells fires in a particular order during a repetitive behavior, then that sequence serves as a template for the reactivation of those episodes in sleep. During replay these cells will still fire in the same relative order independent of the number of measured cells. We augment this approach using phenomenological models (Markov chains) that describe the temporal statistics of the spike avalanches without explicit recourse to how these cells fire during behavior (i.e. independent of the behavioral encoding). These models yield three important observations. First, pyramidal cells are significantly sequentially correlated during sleep and those correlations are enriched for behavior-related sequential firing. Second, these sequential correlations exist between classical place cells (where behavioral sequences are clearly defined) and other pyramidal cells. Third, the sequential structure of bursts during sleep is not simply a ‘noisy’ replay of a singular experience, even in a case where that experience is highly repetitive and salient. Several authors have made similar points before [2,26,27]. We add to this observation by showing that, in fact, the sequential structure of firing during sleep produces many burst words that vary in frequency over several orders of magnitude and including a greater than expected number of long words (Fig 1C, Table 2). The structure of firing during sleep in this way resembles a full language more than a simple mixture of a few noisily repeated patterns. It is an open question for future research behavior modulates this large set of enriched sequences.

## Conclusions

The paradigm of behavioral neuroscience is to relate patterns of neural firing to outcomes in behavioral experiments. Systems neuroscience augments this approach by considering the correlated firing of neuronal ensembles *per se* to gain insight into how behavior shapes neural dynamics and the magnitude of that effect. Using a systems neuroscience approach, we find that repetitive running in a circular track enriches short sequential firing patterns that sum into large sequences of hippocampal pyramidal cells but that these behavior-related patterns are embedded in a much larger network of such short sequences. These sequences include pyramidal cells for which sequential episodic encoding is less straightforward. This shows that sleep replay is fragmented. It coexists and is potentially integrated with many other firing patterns that are likely also to be replay of experiences other than the measured running behavior.

## Materials and Methods

### Animals

3 Sprague Dawley male rats (300–350 g) were used in the study. All behavioral, pharmacological, and surgical procedures were done in accordance with National Institutes of Health guidelines and approved by the Dartmouth College Institutional Care and Use Committee.

### Electrode preparation

32-channel electrode arrays manufactured in the late Robert U. Muller’s Laboratory (State University of New York, Downstate Medical Center, Brooklyn, NY) were used. Seven independently-drivable tetrodes consisting of bundles of 25 μm nichrome wire (A-M Systems, Sequim, WA) were individually inserted into polymicrotubing (Neuralynx, Bozeman, MT). Two additional 100 μm wires (California Fine Wire, Grover Beach, CA) were also included in the electrode array, one used to measure local LFPs and one inserted into the cerebellum as a reference. Two additional stainless steel wires (0.25mm) were soldered to 0.025 cm-thick wire and used for EMG (Plastics One, Roanoke, VA). Tetrodes were connected to a Mill-Max connector (Mill-Max, Oyster Bay, NY) and gold-plated to reduce impedences below 300kΩ.

### Surgery

Rats were anesthetized with isoflurane (2% in O_2_) until unresponsive to a tail pinch. An incision was made in the scalp, skull was exposed and five small holes were drilled. Three skull screws were affixed above the left parietal and left frontal cortex and the cerebellum. The ground wire was soldered to the cerebellar screw and the reference wire was inserted between the skull and the cerebellar parenchyma. The tetrode array was lowered so that the tip of the bundle was above the CA1 pyramidal cell layer (-3.8mm A/P, -2.0mm M/L, -1.8mm D/V). EMG wires were inserted bilaterally into the nuchal muscles. The entire complex was secured to the skull with dental cement. Animals were allowed to recover for a week prior to screening.

### Screening and Data Acquisition

Tetrode assemblies were advanced 50μm three times per day with 4hr between recordings until waveforms of 100μV or larger were detected. The signal from the electrodes was preamplified directly from the rat's head by operational amplifiers and transmitted via a custom cable attached to a rotating commutator connected to a Neuralynx recording system (Neuralynx, Bozeman, MT). Tetrode signal was recorded at 32kHz. EEG was band-pass filtered 0.1-30Hz and EMG signal was filtered at 10-50Hz. Recordings were performed in an open arena placed in a sound proof custom designed Faraday cage. The location of the rat was recorded and tracked using an LED attached to the preamplifier attached to the head of the rat.

### Behavioral Task

Rats (N = 3) were trained to run in a familiar circular track in one direction. The diameter of the circular environment was 1 m, with a 10cm wide path and 60cm high walls. Rats were food restricted and rewarded with a food pellet when turning in the same direction to reinforce the behavior. Sessions where the rat did not turn in the same direction or did not complete the turn were not rewarded and considered incorrect. Animals were required to complete 20 or more correct turns in 15min. Once animals met this criterion, single units and LFPs were recorded during each 20 min session (RUN). After each session, animals were placed back in their home cages and single unit, LFP, and EMG were recorded for 1hr.

### Sleep Scoring

Local EEG data were collected from the hippocampal electrodes and EMG data were collected from leads implanted in the nuchal muscles. EEG was filtered 1–35 Hz and EMG was filtered at 1050 Hz. Theta (4-12Hz) and delta (1-4Hz) power in the EEG signals was calculated. Behavioral states were determined using 10s time windows of EEG and EMG data; awake states were periods of movement on the EMG with a low delta/theta ratio, while sleep states were periods with loss of muscle tone and no activity on the EMG.

### Offline Data Analysis

Action potentials (spikes) were clustered using Neuralynx Spike Sort 3D software (Bozeman, MT). We only retained cells that fired during both RUN and POST. We applied two firing rate filters to select cells for study. First, we only retained cells that fired more than 50 spikes during RUN. Second, to remove interneurons we selected only cells with firing rate less than 10 Hz during POST. Once cells had been selected, firing rate maps were computed in Matlab (MathWorks, Natick, MA) showing action potentials firing per pixel. Rate maps were established by dividing the firing map by the time the animal spent in each pixel. The rate maps showed the number of spikes per pixel per unit time [9,21,28]. Place cells were defined by the spatial localization of their firing fields by two criteria: coherence and concentration of spiking in angular coordinates of the circular track. First, place cell candidates were putative pyramidal cells that had a place field that was more than nine contiguous pixels and a firing rate three or more times greater than the session average [9,21]. Two-dimensional nearest-neighbor autocorrelation of each place cell’s firing rate within a 1x1 cm pixel with its eight nearest neighbors was defined as ‘spatial coherence’ [9,21]. Spatial coherence measures were used as an estimate of the spatial fidelity of cell firing within a place field [9,29]. Second, angular concentration was defined as having > 70% of spikes within a contiguous 40% of the angular distance around the track. Place cells were defined as putative pyramidal cell having coherence > 0.3 and being concentrated in angle as above.

To avoid recording the same cells twice, the tetrodes were advanced at least 25 μm at the end of each recording session. Thus, the neurons sampled in the animals with more than 1 recording were different.

### Spike avalanches and burst word parsing

Spike avalanches are a phenomenon in neural systems where groups of action potentials are fired in bursts and the system becomes quiescent again [7,10–12]. To define the beginning and end of spike avalanches, we first binned the spike trains of the ensemble during sleep. Spike avalanches were then defined as a set of consecutive time bins in which at least one cell had an action potential flanked by time bins during which no cell had an action potential. Because the ensembles varied in numbers of cells, we used a data-driven choice for bin size; the bin size was computed as the mean interspike interval (ISI) for the entire ensemble, i.e. all timestamps for all action potentials of all cells were pooled together [7,13].

The sequence of firing within an avalanche was further parsed into a *burst word* in two steps. First, for a single cell all spikes separated by less than maxISI = 50ms were grouped into a single unit burst represented by the timestamp of the initial spike (c.f. [2]). Next, these time-ordered bursts were converted into a discrete sequence of integers (*symbols*) representing the order of cell firing within the avalanche [2]. We concatenated the words associated with each avalanche by inserting one ‘0’ for each empty time bin between avalanches. This results in a single *burst sequence* of non-negative integers that encodes the sequential behavior of ensemble firing during POST.

### Spike randomization for avalanche null distributions

We computed uncorrelated spike data for use as a null model by independently permuting the interspike intervals (ISIs) for each neuron separately. Specifically, for each neuron we built a random spike train by starting at zero and sampling from the ISI distribution for that neuron without replacement. We then placed a spike at that interval from the previous one and stopped when the ISI distribution was exhausted. Note that this choice of null model by construction preserves the firing rates and ISI distributions (burstiness) of each neuron but breaks all nontrivial correlations between neurons. We performed 100 ISI-randomizations to compute the mean and standard error for the null avalanche size and word length distributions.

We computed the statistical significance of the difference in tails between the null and observed avalanche size distribution using a binomial test. Specifically, we pooled the null size distributions from the 100 ISI-randomizations and defined the large avalanche threshold as the 0.99-quantile of the pooled null. We then counted the number of observed avalanches with size exceeding the threshold and computed the p-value using the binomial cumulative density function. Because the size distribution is discrete, the quantiles jump at each integer. We set the threshold as the last integer to have quantile q < 0.99 and used the corresponding value 1–q as the probability for the binomial test. The test for the word length tails was identical.

### Fitting variable length Markov chains

Below we describe *variable length Markov chains* (VLMCs) in brief to provide relevant mathematical formulae. For a detailed account of the relevant background and inference algorithms, see Begleiter et al. [9].

Let *S* = {0,1,2,…,*C*} be the set of symbols representing each of C cells and the silence character in the burst sequence and let $\vec{s}=s_{1}s_{2}\ldots s_{N}$ be a sequence of length N. For any Markov chain over the alphabet S, the probability of the sequence s is given by

$$P_{M}(\vec{s})=\prod_{i=1}^{N}P_{M}(s_{i}|s_{1}s_{2}\ldots s_{i-1}),$$

i.e. the probability of the upcoming symbol s_i_ is a function of the history of the sequence. Typically these conditional probabilities only depend explicitly on the most recent symbols and the amount of relevant memory can vary in length (see below and [9] for an extended discussion).

The *log-loss* of a model M in predicting a sequence s is given by

$${\text{L}}_{M}(\vec{s})=-\frac{1}{N}\sum_{i=1}^{N}{\text{log}}_{2}\left(P_{M}(s_{i}|s_{1}s_{2}\ldots s_{i-1})\right)$$

The model M is a good fit for the sequence s if the log-loss is small; the log-loss is small if the probability of each symbol is high given the context. The log-loss function is a goodness-of-fit measure for Markov chain models. Log-loss is measured in bits.

We fit variable length Markov chain models (VLMCs) to the burst sequence using the Matlab software provided by Begleiter et al. as a companion to the paper [9]. Specifically, we used the Probabilistic Suffix Tree (PST) algorithm. In brief, the PST algorithm fits a VLMC model in two stages: first it filters possible contexts (up to a given maximum depth) by occurrence frequency in the data, then it tests how informative the contexts by iteratively considering the predictive power of longer contexts relative to shorter contexts contained within, i.e. it statistically compares the difference in predictive power of longer contexts compared to shorter ones.

The PST algorithm has 4 user parameters. Most importantly, the maximum depth parameter, d, defines the maximum amount of sequential memory used for predicting the future, i.e. the maximum context length allowed by the VLMC model. We fit VLMC models for depths d = 0, 1,…,5 and chose d by 10-fold cross validation selecting the value of d that produced the lowest average log-loss over the 10 testing folds (Fig 2A).

In addition to the maximum depth, the PST algorithm has hyperparameters defining the filtering stages of context identification. The PST algorithm only considers contexts that occur above a (small) minimum frequency, P_min_, within the data. Furthermore, for a each context, σ, there must exist a possible future symbol, s, for which the probability of transitioning to that symbol, P(s|σ), exceeds a (small) user-defined threshold, γ. In addition, letting σ’ be the suffix of σ obtained by shortening by one symbol, the PST algorithm requires that the ratio of probabilities P(s|σ) / P(s|σ’) (or its inverse) exceed a user-defined threshold r > 1. These last two parameters are used to prune the context tree and they ensure that the algorithm is only learning contexts that are relevant in the training data. We set P_min_ = 0.006, γ = 0.001, and r = 1.05. Manual tuning showed that the results were relatively robust to these choices.

### Sequential information and normalization

We fit depth-d models to each burst sequence for d = 0,…,5. Let L_d_ be the average log-loss of the depth-d model over the 10 cross-validation rounds and let L_opt_ the minimum average log-loss over all tested values of d. The difference, *I*_*d*_ = *L*_0_ − *L*_*d*_, between the unstructured 0^th^-order model and the depth-d model is a measure of the amount of sequential structure captured by the depth d model. We call I_d_ the *sequential information*. We define I_opt_ analogously. Like log-loss, I_d_ is measured in bits. To make comparisons between animals and sessions, we define the *normalized sequential information*, $NI_{d}=\frac{I_{d}}{I_{opt}}$. NI varies between 0, for a model with no learned sequential structure, and 1, for the optimal model.

### The avalanche independent model and sequence likelihood ratios

A sequence can either be enriched or depleted by the temporal structure of the fitted VLMC model meaning that it occurs at a rate higher or lower than in a ‘background model’. An obvious choice for background model is to use the fully independent model of independent, identically distributed random draws from the set of symbols in the data sequence. This model has no sequential structure at all and therefore breaks the avalanche structure of the burst sequence, which produces longer words and longer periods of silence than expected under the fully independent model. An alternative background model we call the *avalanche independent model*, AIM, has no sequential correlations between neurons, but does preserve the avalanche structure.

We define the AIM to be the first-order Markov chain that preserves the rates of transition between silence and bursting but has no other structure. Specifically, we compute rates of transitions from silence to silence (0–0 in the sequence), silence to bursting (0-i, for some cell i), bursting to silence (i-0), and bursting to bursting (i-j). Denote these by P_00_, P_0b_, P_b0_, and P_bb_ respectively. Furthermore, let P_1_, P_2_,…, P_C_ be the burst rates of each cell normalized by the total number of bursts. The transition matrix of the burst independent model is then given by

$$\begin{array}{l} P_{AI}(0|0)=P_{00}\\ P_{AI}(i|0)=P_{0b}P_{i}\\ P_{AI}(0|i)=P_{b0}\\ P_{AI}(i|j)=P_{i} \end{array}$$

Note in particular that there is no sequential structure between bursts (last line). The avalanche independent model serves as a comparator for sequentially structured models that have the same transition rates to and from silence.

We define the *likelihood ratio* for a sequence $\vec{s}$ as the ratio

$$L(\vec{s})=\frac{P(\vec{s})}{P_{AI}(\vec{s})},$$

where P is the fitted model. Let $l(\vec{s})={\text{log}}_{2}\left(L(\vec{s})\right)$ denote the log-likelihood ratio.

Because we cross-validated when we fit P, we compute statistical significance of likelihood ratios by standardizing the log-likelihood ratios. Let $\vec{s}$ be a sequence (e.g. a RUN sequence) and let $l_{i}(\vec{s})$ be the log-likelihood ratio for the i^th^ cross-validation fold. Let 〈⋅〉 denote averaging over i = 1,…,10. We define the z-score of a sequence as

$$z(\vec{s})=\frac{\langle l_{i}(\vec{s})\rangle }{\sqrt{\langle l_{i}{\left(\vec{s}\right)}^{2}\rangle -{\langle l_{i}(\vec{s})\rangle }^{2}}}\;,$$

i.e. the mean log-likelihood divided the standard deviation over the 10 cross-validation folds. We then compute a p-value by comparing the z-score to the standard normal distribution.

### Conditional log-likelihood ratios and enriched transitions

A Markov chain is a conditional probability table. It defines transition probabilities *given the current context*. We wish to compare transition probabilities between two models, the fitted model and the AIM to discern which transitions occur more/less than expected in the fitted model. To do this, we define *conditional log-likelihood ratios*.

Let Σ = {*σ*_1_, *σ*_2_,…,*σ*_*K*_} be the set of contexts for the fitted VLMC model. We define the *conditional log-likelihood matrix* R as

$$R(\sigma_{i},j)={\text{log}}_{2}\left(\frac{P(j|\sigma_{i})}{P_{AI}(j|\sigma_{i})}\right)$$

The matrix R is rectangular with rows indexed by the contexts of the fitted VLMC model and columns indexed by cells (and ‘0’). R encodes the up- and down-regulation of the background bursting rates as a function of context. The logarithm treats up- and down-regulation on the same scale.

To visually inspect the structure of these matrices, we clustered R using cosine similarity and average linkage hierarchical clustering using the Matlab function ‘linkage’. We drew cluster boundaries (Fig 6, black lines) using greedy modularity maximization[30]. We note that these cluster boundaries are only for visualization.

### RUN-optimal Markov chains

To assess the capacity of the measured burst rates to produce the RUN sequence, we constructed first-order Markov chains that produced this word at the highest rate possible given the measured burst rates. We computed a *RUN-optimal Markov chain* by optimizing the transition probabilities of the chain to maximize the rate of producing the RUN sequence. The stationary distribution of the chain equal the long-run burst rates measured in the data and these are a function of all transition probabilities, not just those in the RUN sequence. We constrained the optimization so that the solution would have the measured burst rates. To preserve the avalanche structure of the data, we constrained the silence-to-silence transition rate to equal that measured in the data sequence (as we did with the AIM above). These are linear constraints on the transition matrix. We used the nonlinear optimization function ‘fmincon’ in Matlab to perform the optimization.

## Supporting Information

S1 Fig Spatial variation of pyramidal cells firing during RUN. Spatial rates maps for rat 1, session 1.
(TIFF)

S2 Fig Spatial variation of pyramidal cells firing during RUN. Spatial rates maps for rat 1, session 2.
(TIFF)

S3 Fig Spatial variation of pyramidal cells firing during RUN. Spatial rates maps for rat 3, session 1.
(TIFF)

S4 Fig Spatial variation of pyramidal cells firing during RUN. Spatial rates maps for rat 3, session 2.
(TIFF)

S5 Fig Clustering of VLMC contexts. Cluster analysis of VLMC contexts for rat 1, session 1.
(TIFF)

S6 Fig Clustering of VLMC contexts. Cluster analysis of VLMC contexts for rat 2.
(TIFF)

S7 Fig Clustering of VLMC contexts. Cluster analysis of VLMC contexts for rat 3, session 1.
(TIFF)

S8 Fig Clustering of VLMC contexts. Cluster analysis of VLMC contexts for rat 3, session 2.
(TIFF)

## References

[1] Skaggs WE, McNaughton BL. Replay of neuronal firing sequences in rat hippocampus during sleep following spatial experience. Science. 1996;271: 1870–1873. 8596957 10.1126/science.271.5257.1870

[2] Lee AK, Wilson MA. Memory of sequential experience in the hippocampus during slow wave sleep. Neuron. 2002;36: 1183–1194. 12495631 10.1016/s0896-6273(02)01096-6

[3] Diba K, Buzsáki G. Forward and reverse hippocampal place-cell sequences during ripples. Nat Neurosci. 2007;10: 1241–1242. 10.1038/nn1961 17828259 PMC2039924

[4] Foster DJ, Wilson MA. Reverse replay of behavioural sequences in hippocampal place cells during the awake state. Nature. 2006;440: 680–683. 10.1038/nature04587 16474382

[5] Pfeiffer BE, Foster DJ. Hippocampal place-cell sequences depict future paths to remembered goals. Nature. 2013;497: 74–79. 10.1038/nature12112 23594744 PMC3990408

[6] John M Beggs NT. Being Critical of Criticality in the Brain. Frontiers in Physiology. Frontiers Media SA; 2012;3: 163 10.3389/fphys.2012.00163 22701101 PMC3369250

[7] Ribeiro TL, Copelli M, Caixeta F, Belchior H, Chialvo DR, Nicolelis MAL, et al Spike Avalanches Exhibit Universal Dynamics across the Sleep-Wake Cycle. PLoS ONE. Public Library of Science; 2010;5: e14129 10.1371/journal.pone.0014129 21152422 PMC2994706

[8] Beggs JM, Plenz D. Neuronal avalanches in neocortical circuits. The Journal of neuroscience. 2003. 10.1523/JNEUROSCI.23-35-11167.2003 PMC6741045 14657176

[9] Begleiter R, El-Yaniv R, Yona G. On Prediction Using Variable Order Markov Models. Journal of Artificial Intelligence Research. 2004;: 385–421.

[10] Bostock E, Muller RU, Kubie JL. Experience-dependent modifications of hippocampal place cell firing. Hippocampus. 1991;1: 193–205. 10.1002/hipo.450010207 1669293

[11] Anderson MI, Jeffery KJ. Heterogeneous modulation of place cell firing by changes in context. Journal of Neuroscience. 2003;23: 8827–8835. 14523083 10.1523/JNEUROSCI.23-26-08827.2003 PMC6740394

[12] Jeffery KJ, Gilbert A, Burton S, Strudwick A. Preserved performance in a hippocampal-dependent spatial task despite complete place cell remapping. Hippocampus. 2003;13: 175–189. 10.1002/hipo.10047 12699326

[13] Harris KD, Csicsvari J, Hirase H, Dragoi G, Buzsáki G. Organization of cell assemblies in the hippocampus. Nature. 2003;424: 552–556. 10.1038/nature01834 12891358

[14] Buzsáki G. Neural Syntax: Cell Assemblies, Synapsembles, and Readers. Neuron. Elsevier Inc; 2010;68: 362–385. 10.1016/j.neuron.2010.09.023 21040841 PMC3005627

[15] Harris KD. Neural signatures of cell assembly organization: Abstract: Nature Reviews Neuroscience. Nat Rev Neurosci. 2005. 10.1038/nrn1669 15861182

[16] Battaglia FP, Benchenane K, Sirota A, Pennartz CMA, Wiener SI. The hippocampus: hub of brain network communication for memory. Trends Cogn Sci (Regul Ed). 2011;15: 310–318. 10.1016/j.tics.2011.05.008 21696996

[17] Frankland PW, Bontempi B. The organization of recent and remote memories. Nat Rev Neurosci. 2005;6: 119–130. 10.1038/nrn1607 15685217

[18] Mehta MR. Cortico-hippocampal interaction during up-down states and memory consolidation. Nat Neurosci. 2007;10: 13–15. 10.1038/nn0107-13 17189946

[19] Dupret D, O'Neill J, Pleydell-Bouverie B, Csicsvari J. The reorganization and reactivation of hippocampal maps predict spatial memory performance. Nat Neurosci. 2010;13: 995–1002. 10.1038/nn.2599 20639874 PMC2923061

[20] Wamsley EJ. Dreaming and offline memory consolidation. Curr Neurol Neurosci Rep. 2014;14: 433 10.1007/s11910-013-0433-5 24477388 PMC4704085

[21] Muller RU, Kubie JL, Ranck JB. Spatial firing patterns of hippocampal complex-spike cells in a fixed environment. J Neurosci. 1987;7: 1935–1950. 3612225 10.1523/JNEUROSCI.07-07-01935.1987 PMC6568929

[22] Brown EN, Frank LM, Tang D, Quirk MC, Wilson MA. A statistical paradigm for neural spike train decoding applied to position prediction from ensemble firing patterns of rat hippocampal place cells. J Neurosci. 1998;18: 7411–7425. 9736661 10.1523/JNEUROSCI.18-18-07411.1998 PMC6793233

[23] Zhang K, Ginzburg I, McNaughton BL, Sejnowski TJ. Interpreting neuronal population activity by reconstruction: unified framework with application to hippocampal place cells. J Neurophysiol. 1998;79: 1017–1044. 9463459 10.1152/jn.1998.79.2.1017

[24] Davidson TJ, Kloosterman F, Wilson MA. Hippocampal replay of extended experience. Neuron. 2009;63: 497–507. 10.1016/j.neuron.2009.07.027 19709631 PMC4364032

[25] Bendor D, Wilson MA. Biasing the content of hippocampal replay during sleep. Nat Neurosci. 2012;15: 1439–1444. 10.1038/nn.3203 22941111 PMC4354843

[26] Buhry L, Azizi AH, Cheng S. Reactivation, Replay, and Preplay: How It Might All Fit Together. Neural Plasticity. 2011;2011: 1–11. 10.1152/jn.00897.2006 PMC3171894 21918724

[27] Gupta AS, van der Meer MAA, Touretzky DS, Redish AD. Hippocampal Replay Is Not a Simple Function of Experience. Neuron. Elsevier Ltd; 2010;65: 695–705. 10.1016/j.neuron.2010.01.034 20223204 PMC4460981

[28] Lenck-Santini P-P, Muller RU, Save E, Poucet B. Relationships between place cell firing fields and navigational decisions by rats. Journal of Neuroscience. Society for Neuroscience; 2002;22: 9035–9047. 12388610 10.1523/JNEUROSCI.22-20-09035.2002 PMC6757700

[29] Zhou J-L, Lenck-Santini P-P, Zhao Q, Holmes GL. Effect of interictal spikes on single-cell firing patterns in the hippocampus. Epilepsia. 2007;48: 720–731. 10.1111/j.1528-1167.2006.00972.x 17284294

[30] Newman MEJ. Modularity and community structure in networks. Proc Natl Acad Sci USA. 2006;103: 8577–8582. 10.1073/pnas.0601602103 16723398 PMC1482622

