# Supplementary figures and images for: Short-Range Temporal Interactions in Sleep; Hippocampal Spike Avalanches Support a Large Milieu of Sequential Activity Including Replay
Source: PLoS One. 2016 Feb 11;11(2):e0147708. doi: 10.1371/journal.pone.0147708 (PMC4750866; doi:10.1371/journal.pone.0147708)

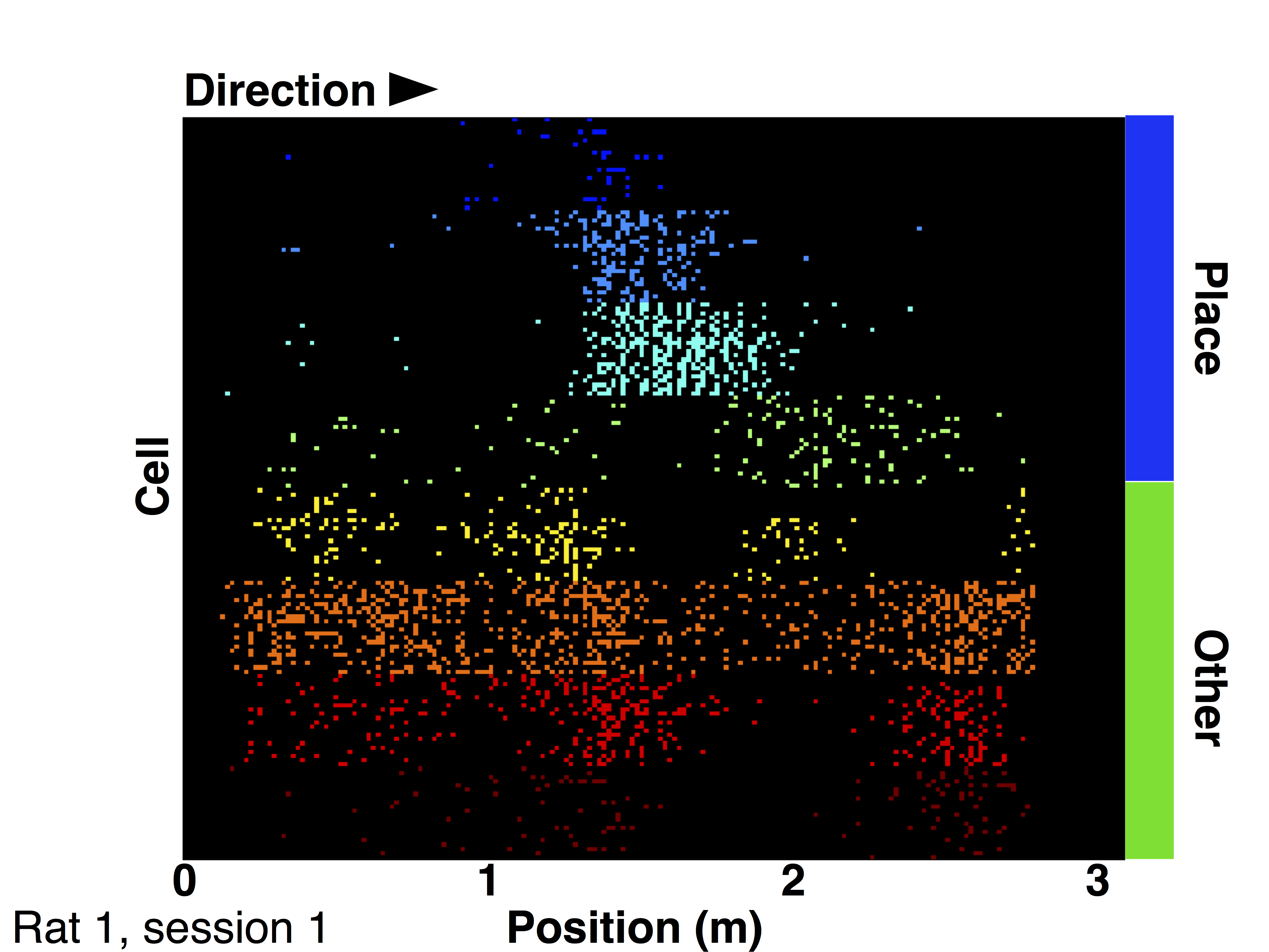

Supplement: S1 Fig — Spatial rates maps for rat 1, session 1. (TIFF) [file pone.0147708.s001.tiff]

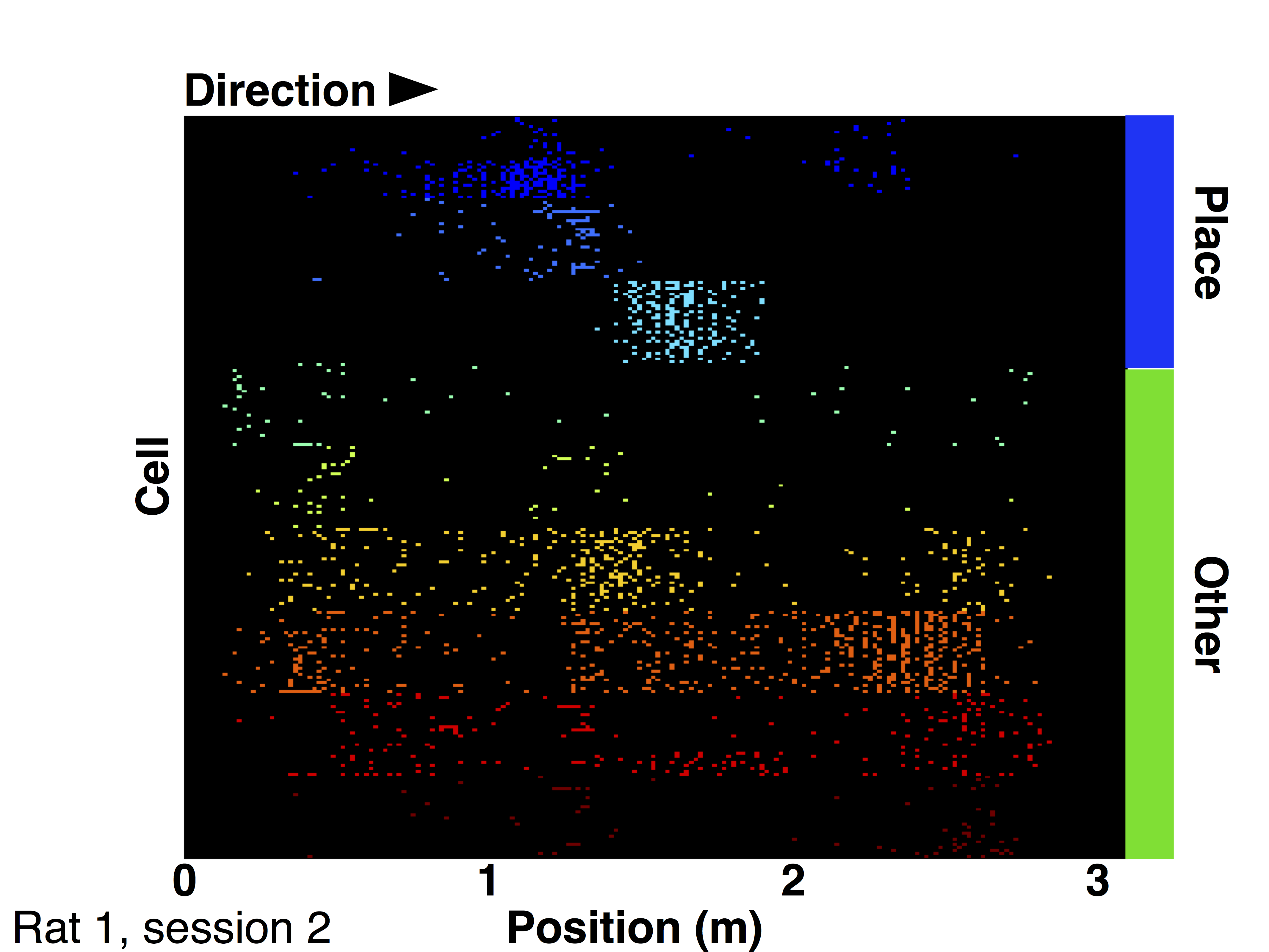

Supplement: S2 Fig — Spatial rates maps for rat 1, session 2. (TIFF) [file pone.0147708.s002.tiff]

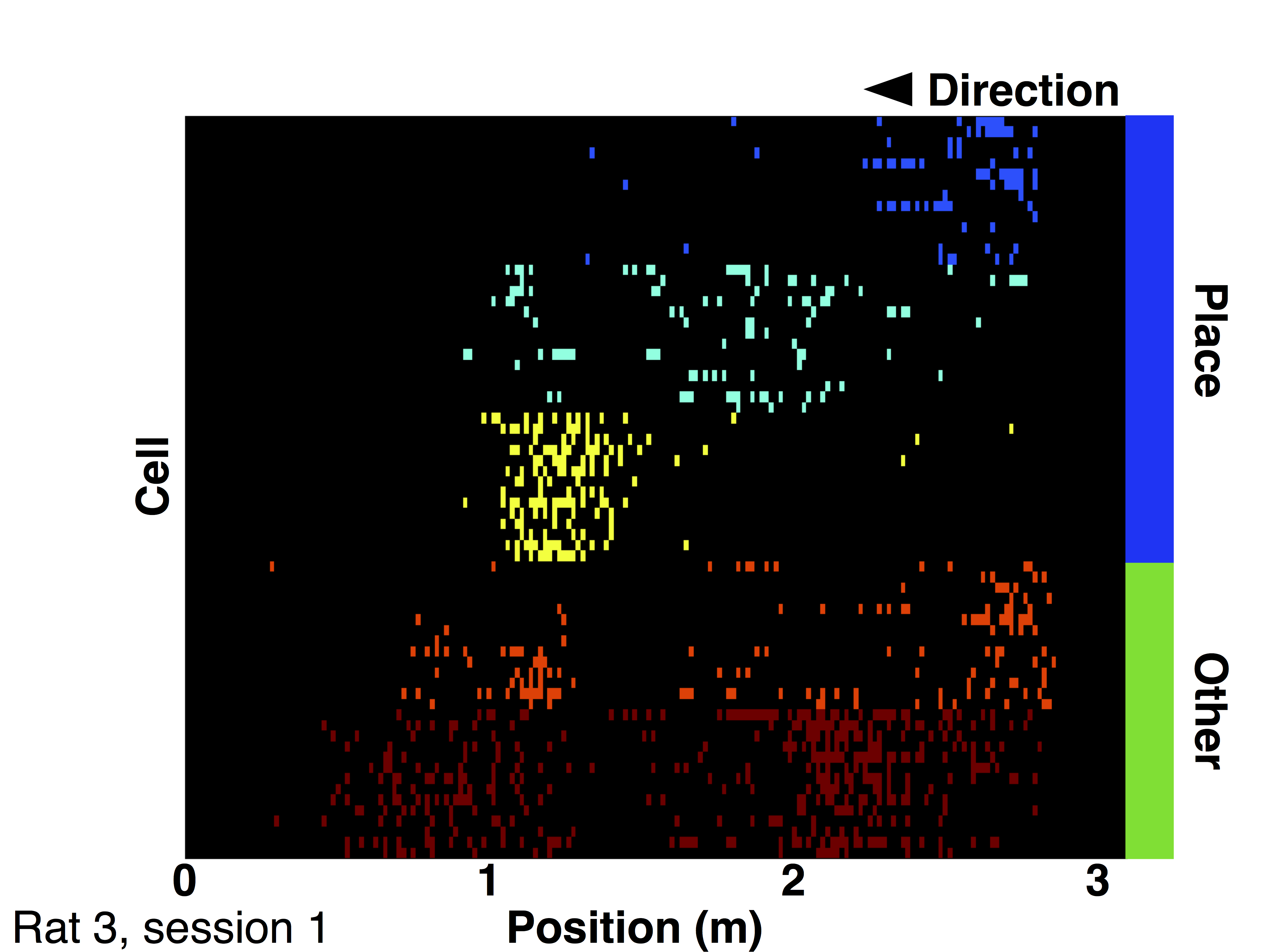

Supplement: S3 Fig — Spatial rates maps for rat 3, session 1. (TIFF) [file pone.0147708.s003.tiff]

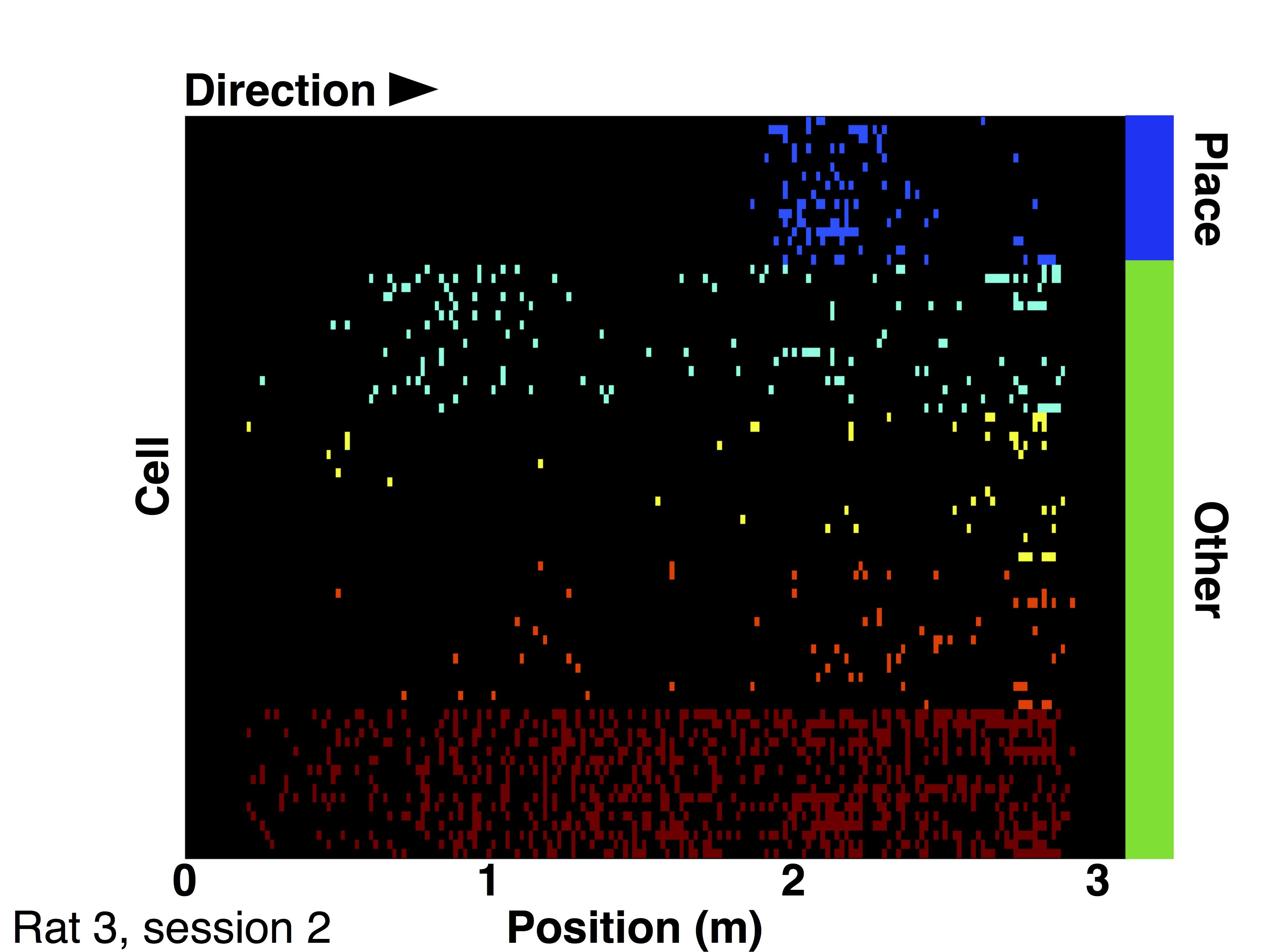

Supplement: S4 Fig — Spatial rates maps for rat 3, session 2. (TIFF) [file pone.0147708.s004.tiff]

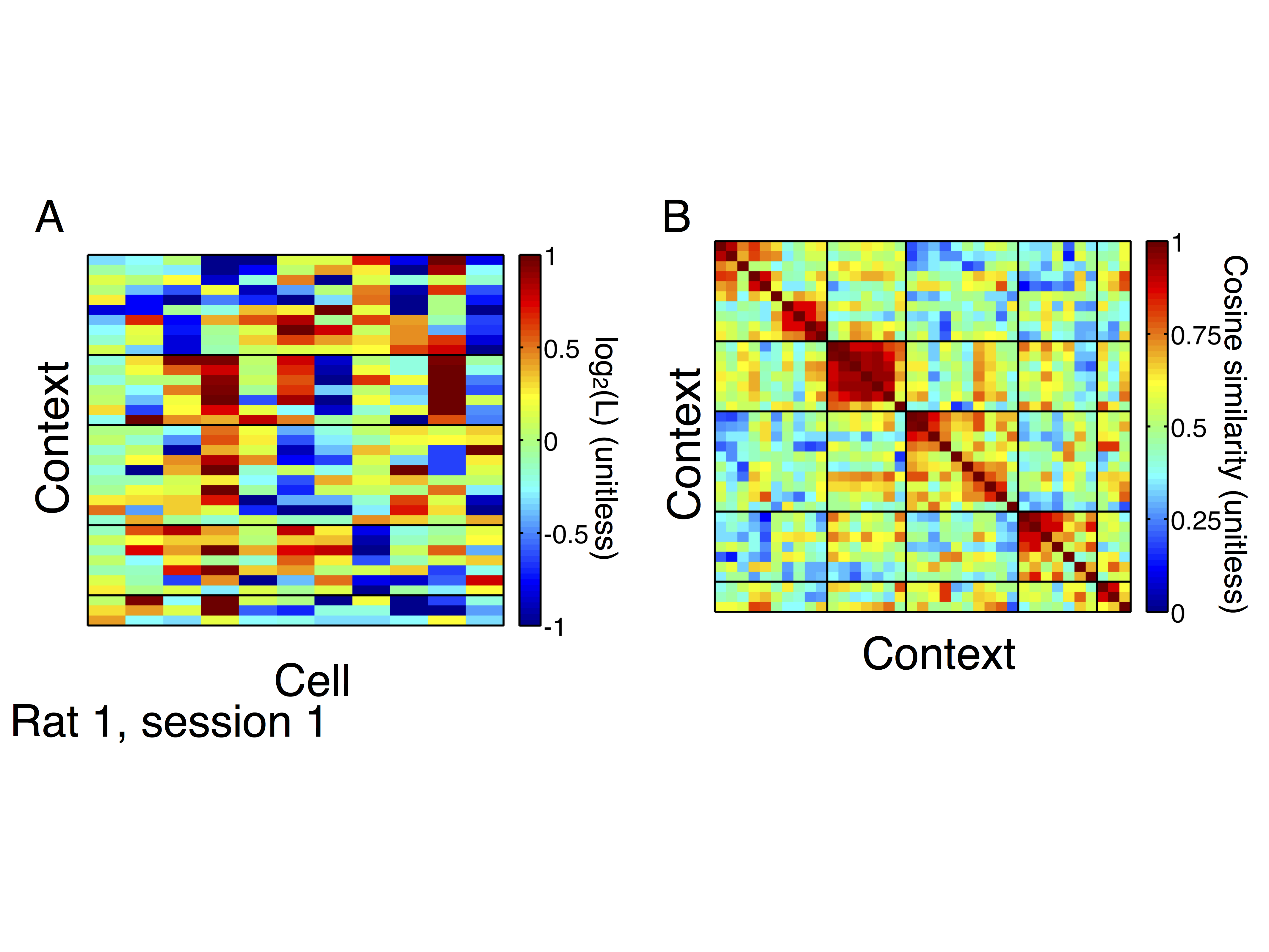

Supplement: S5 Fig — Cluster analysis of VLMC contexts for rat 1, session 1. (TIFF) [file pone.0147708.s005.tiff]

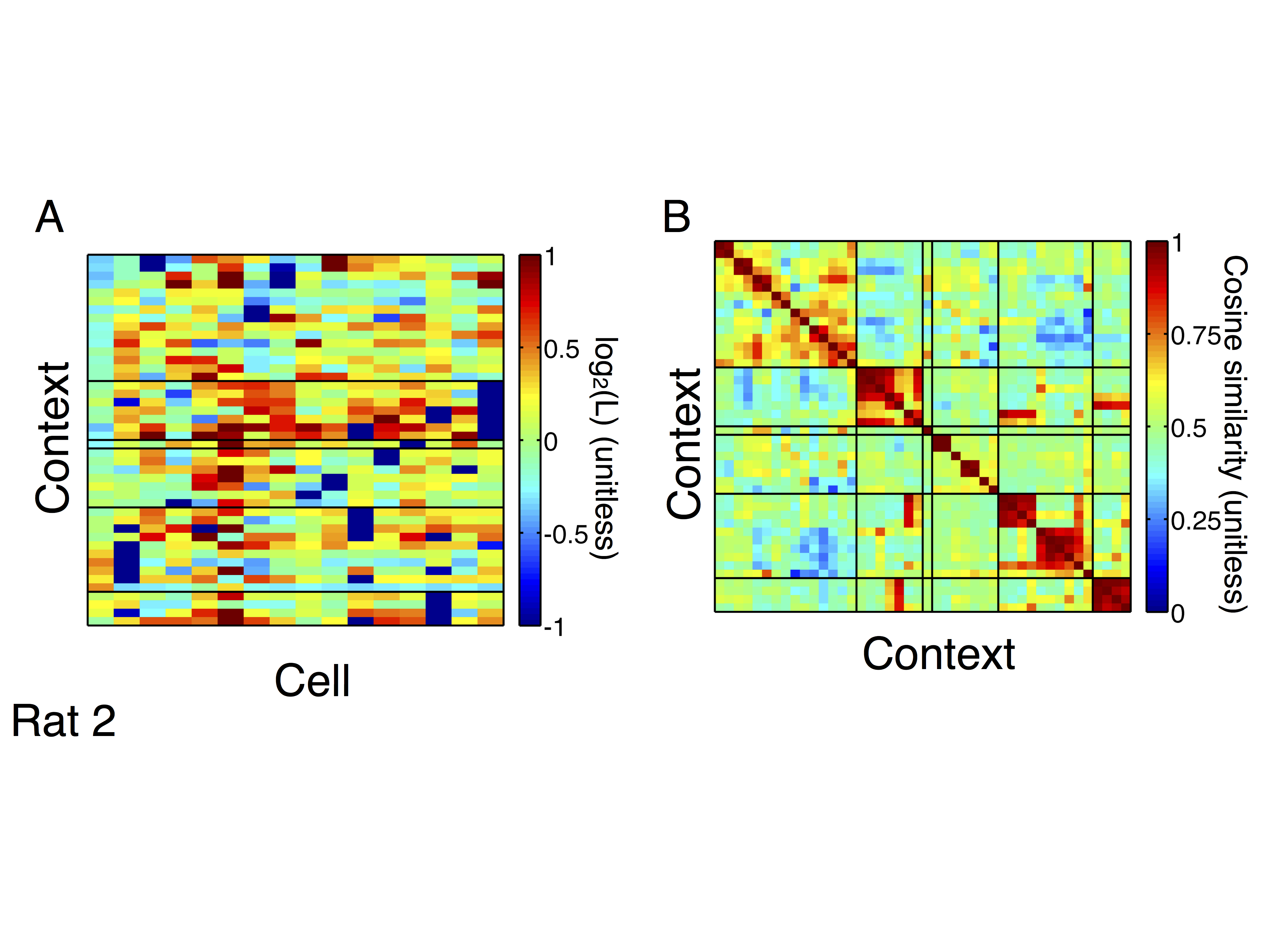

Supplement: S6 Fig — Cluster analysis of VLMC contexts for rat 2. (TIFF) [file pone.0147708.s006.tiff]

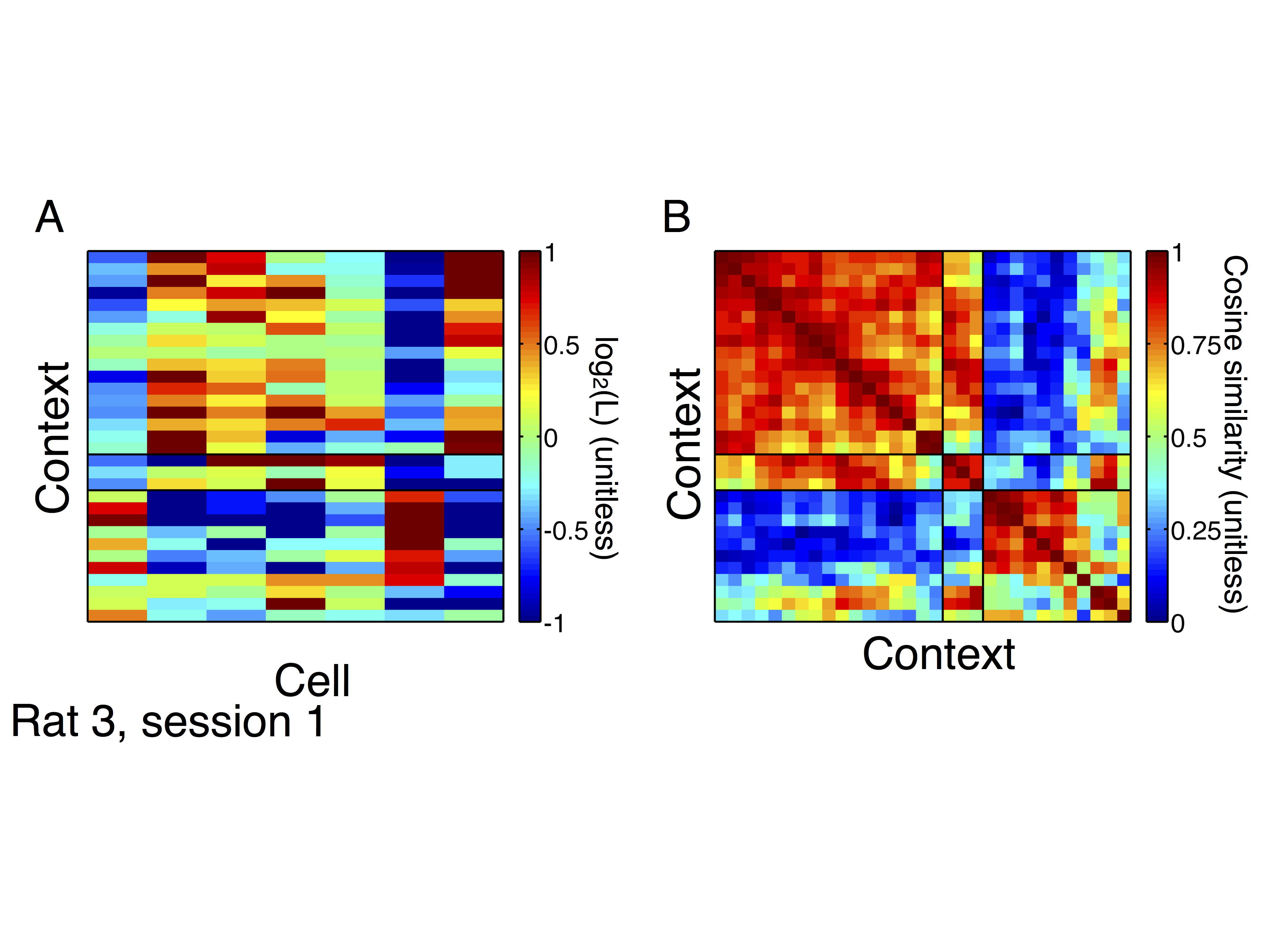

Supplement: S7 Fig — Cluster analysis of VLMC contexts for rat 3, session 1. (TIFF) [file pone.0147708.s007.tiff]

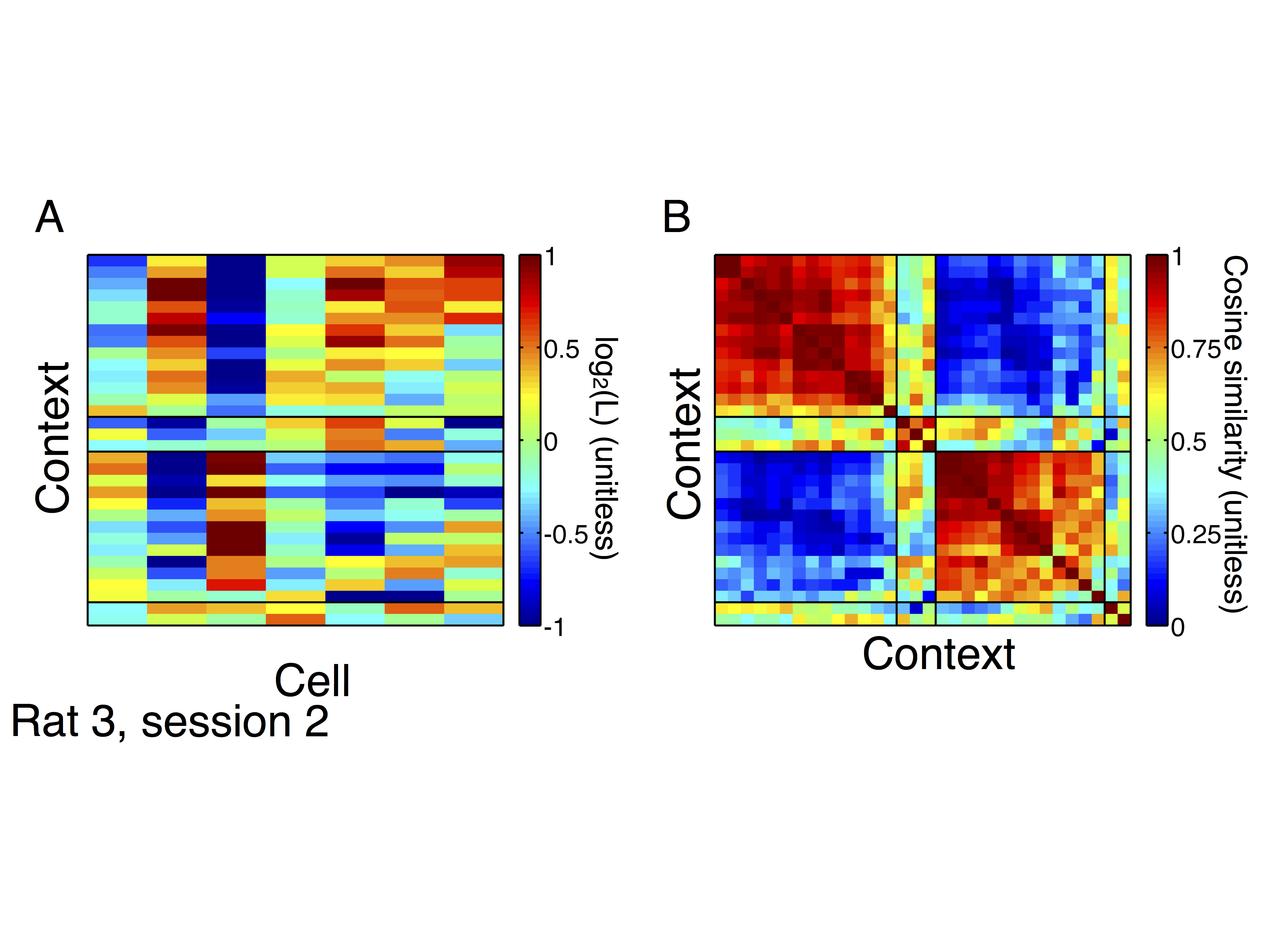

Supplement: S8 Fig — Cluster analysis of VLMC contexts for rat 3, session 2. (TIFF) [file pone.0147708.s008.tiff]
